# Managing the work stress of inpatient nurses during the COVID-19 pandemic: a systematic review of organizational interventions

**DOI:** 10.1186/s12912-024-02358-1

**Published:** 2024-09-27

**Authors:** Maria Zink, Frederike Pischke, Johannes Wendsche, Marlen Melzer

**Affiliations:** 1 Federal Institute for Occupational Safety and Health (BAuA), Dresden, Germany; 2 Technische Universität Dresden (TUD), Dresden, Germany

**Keywords:** COVID-19 pandemic, Hospital, Inpatient care, Inpatient nurse, Nursing homes, Organizational workplace intervention, Systematic review, Work organization

## Abstract

**Background:**

During the coronavirus disease 2019 (COVID-19) pandemic, inpatient nurses faced various work stressors. Little is known about organizational interventions that can mitigate the negative consequences of pandemic-related stressors.

**Objective:**

The aim was to provide a synopsis of the literature concerning the types and outcomes of organizational interventions performed during the COVID-19 pandemic that directly (re)organized the work structures of inpatient nurses to address pandemic-related work stressors or to increase nurses’ ability to cope.

**Methods:**

Within this preregistered systematic literature review, we searched four databases (PubMed, PsycINFO, PsycARTICLES, CINAHL) and two preprint databases (MedRxiv, PsyArXiv) for interventional studies of organizational interventions published between 01/2020 and 03/2023 (k = 990 records). We included 12 primary studies after title-abstract and full-text screening. A synthesis of results without meta-analysis was conducted. Risk of bias was assessed with the Cochrane risk-of-bias tool for randomized trials – version 2 (RoB-2) and Risk Of Bias In Non-randomized Studies - of Interventions (ROBINS-I) tool.

**Results:**

All interventions were implemented in hospitals. The reasons given for implementation included pandemic-related work stressors such as a high workload, understaffing, and a lack of medical resources. To respond to the various work stressors, half of the studies took a multilevel approach combining organizational and person-oriented interventions (k = 6). Most studies (k = 8) took a secondary prevention approach, focusing on the organization of rest breaks (k = 5). With respect to outcomes, the studies examined nurse-related stress and resilience, turnover intention, job satisfaction, and other factors. Risk-of-bias analyses revealed that conclusions about the effectiveness of the interventions are limited due to confounding factors and self-selection.

**Conclusions:**

The identified interventions provide a basis for future research to draw conclusions on the effectiveness of organizational interventions during pandemics. The promotion of adequate work breaks could be useful if the work stressors associated with strain and negative consequences cannot be changed directly. However, the same stressors (e.g., high workload) can hinder nurses from participating in offered interventions. This emphasizes the importance of directly addressing inpatient nurses’ work stressors.

**Registration:**

Prospero-ID CRD42023364807 (March 2023).

**Supplementary Information:**

The online version contains supplementary material available at 10.1186/s12912-024-02358-1.

## Background

The coronavirus disease 2019 (COVID-19) pandemic has demonstrated the incremental systemic importance of nurses and the effectiveness of the healthcare system for society. Moreover, nurses’ working conditions and the professional challenges faced by nurses in delivering high-quality patient care—often with constrained resources and frequent ethical dilemmas—are sources of high levels of work stress [1]. Working conditions that reduce work stress and allow nursing staff to remain healthy in times of crisis is particularly important to ensure high-quality health care.

The COVID-19 pandemic exacerbated the already suboptimal working conditions in inpatient care settings; among all health care workers, nurses suffered the most from pandemic-related work stressors [2]. Specifically, nurses working with COVID-19 inpatients experienced direct consequences of the pandemic: high infection risks (two times greater than that of non-essential workers in Germany [3]; and almost four times greater than that of the general community in the UK and USA; [4]), the suffering of patients and patient caregivers, and death and isolation [2]. Additionally, inpatient nurses faced changes in their working conditions, such as staff shortages, high workloads, long work shifts, a lack of breaks and personal protective equipment (PPE), and social stigma [5, 6]. However, little is known about which organizational measures can prevent such stressors or at least mitigate their negative effects.

The need for action arises when the consequences of the aforementioned work stressors for nurses, patients, and inpatient facilities (that is, hospitals or nursing homes) during the pandemic are considered. Several meta-analyses revealed that high levels of pandemic-related work stress are related to a greater risk of physical strain (e.g., skin irritation from continuously wearing PPE), mental strain (e.g., depression, anxiety, emotional exhaustion) and negative organizational outcomes (e.g., patient safety and high turnover risks) [5, 7–10].

To prevent such negative consequences, organizations (hospitals, nursing homes, etc.) adopted early interventions focusing predominantly on relieving individual stress-related symptoms [11]. Individual-level interventions, as part of the person-oriented approach, aim to influence employee behavior, coping strategies, and strain reactions (e.g., fatigue) in response to work stressors. Systematic reviews have shown that such interventions improved nurses’ resilience and ability to cope with work stressors during the COVID-19 pandemic [12, 13]. However, Muller et al. [11] noted that there is a mismatch between ‘organizational sources of psychological distress, such as workload and lack of PPE, and how healthcare systems are attempting to relieve distress at an individual level’ (p. 8). Therefore, there is a strong need for a human-centered work design that ensures nurses’ well-being. This can be achieved by interventions that take a job-oriented approach that aims to modify work stressors themselves. Interventions based on the job-oriented approach aim to permanently change working conditions to address sources of adverse effects or provide resources to reduce these effects [14, 15].

Focusing on the job-oriented approach and thus directly influencing the work design of inpatient settings has stronger sustainable effects on employee health than individual-level interventions considering the person-oriented approach [16]. Furthermore, the job-oriented approach is important for primary and secondary prevention. A review of earlier pandemics and epidemics supported the belief of Muller et al. [11]. Kisely et al. [17] who identified organizational factors such as transparent and supportive communication, rest breaks, personal health protection, and practical support as important components of primary prevention interventions. The job-oriented approach is also relevant in the context of secondary prevention interventions: by directly changing working conditions (for example, by providing work breaks), inpatient nurses can be supported in coping with stressful working conditions that cannot be changed directly (e.g., a high COVID-19 patient load). In conclusion, interventions that take a job-oriented approach could either modify adverse work stressors themselves (primary prevention) or change working conditions to provide buffering resources or reduce adverse short-term strain outcomes (secondary prevention).

To address the work stressors and strain that arose throughout the COVID-19 pandemic through interventions that take a job-oriented approach, it is important to consider the complexity of the sociotechnical work system of inpatient nurses [18]. The Systems Engineering Initiative for Patient Safety 2.0 (SEIPS 2.0; [19]) model considers the work system of inpatient nurses during the COVID-19 pandemic [20]. This is due to its explicit inclusion of the external environment; its ability to distinguish patient, professional, and organizational outcomes; and its recent wide use in nursing systems research.

The SEIPS 2.0 model [19] proposes that work stressors comprise six interacting components: *persons*,* tasks*,* tools and technologies*,* organization*, and the *internal* and *external environment*. In light of the objective of this review, the organization and the external environment components are briefly explained in the following paragraphs.

The *organization component* includes all external structures and can also refer to the physical environment, time, resources, and activities. Consequently, the organization of activities includes the task component of the work system. Similarly, resources are related to tools and technologies, and the physical environment is related to the internal environment. Additionally, social organization is seen as part of the organization component. The organization component was particularly important in this review, as it is related to the work system by means of work organization (i.e., searching for interventions that reorganize the external structures of nurses) [19].

The *external environment* of the work system represents macrolevel factors outside an organization, such as ecological, societal, and policy factors. During the COVID-19 pandemic, external factors included, for example, high infection rates associated with higher hospitalization rates or political regulations regarding infection control and infection prevention that influenced inpatient nurses’ work behavior [6].

By taking a job-oriented approach, in the present review, we searched for and analyzed interventions that were implemented at an organizational level and carried out during the COVID-19 pandemic. Organizational interventions were defined as prospectively planned interventions that directly shaped the work content and/or work context in a ‘top-down’ manner. This included the (re)design of work system components outside the individual person, such as work activities (e.g., quantity of work tasks), work resources (e.g., technologies, human resources), the physical work environment (e.g., lighting, unit organization), and temporal (e.g., work schedules) or social (e.g., mentorships) aspects, according to the SEIPS 2.0 categories for work organization.

To date, only the review by Nicolakakis et al. [21] has explicitly addressed the effectiveness of organizational interventions published up to 2021. However, they considered organizational interventions during pandemics or epidemics in general while limiting their search for studies reporting mental no studies on comparable interventions seven studies, with all studies (k = 5 [22–26]), referring to the COVID-19 pandemic being observational. Within these five studies, the interventions ranged from interventions involving leadership training and the provision of peer support and rest breaks to simulation-based interventions. Interventions that changed multiple work aspects, offered psychological support (from peers or nurse leaders) or integrated participatory elements to tailor the intervention to nurses’ needs were identified as helpful in reducing nurses’ work stress and improving their mental health. Owing to poor study quality, the authors expressed low confidence in their effectiveness and the need for better-designed studies with good implementation strategies. A common problem of the five identified studies was the failure to address the core issue of nurses’ stress or methodological issues [21].

In this systematic review, we updated the findings of Nicolakakis et al. [21] without restricting our search to specific outcomes and focusing on studies with an interventional design. Therefore, we expanded our knowledge of evidence-based interventions that aim to directly change workplace aspects that can cause strain or hinder (for example, high workloads) or support (for example, adequate rest breaks) inpatient nurses’ ability to cope with high job demands. Therefore, our core research question, which followed the PICOS framework, was as follows:

*Which organizational interventions (I) during the COVID-19 pandemic addressed pandemic-associated work stressors among inpatient nurses (P) in interventional studies (S)*,* and what were the outcomes of these interventions (O) compared with those of no or other interventions (C)?*

Following the general research question, further questions were derived from the findings and theoretical arguments presented above. Therefore, the considerations by Nielsen and Noblet [27] regarding the question ‘what works for whom?’ and the Template for Intervention Description and Replication (TIDieR) checklist [28] were considered.

1. What pandemic-associated work stressors were addressed by intervention studies during the COVID-19 pandemic?
2. Which organizational level interventions implemented what materials/procedures in what way to address work stressors of inpatient nurses during the COVID-19 pandemic?
3. Did the included studies modify the adverse work stressors themselves (primary prevention) or change work aspects to buffer the negative effects (secondary prevention)?
4. Which types of components of the work system (activities, resources, physical environment, temporal or social aspects) were modified, and how often were they modified?
5. Were the organizational interventions combined with other (e.g., person-oriented) interventions?
6. What patient, professional and/or organizational outcomes of the organizational interventions implemented to address the COVID-19 pandemic-associated work stressors of inpatient nurses were reported by the included studies?
7. What hindrances or promoting factors were discussed in the included intervention studies?

Question 1 considered the circumstances and the rationale for why the intervention was implemented. Questions 2, 3, 4 and 5 aimed to describe the interventions in more detail. Questions 6 and 7 provided information on the outcome and ‘how well and why’ an intervention worked or did not work.

## Methods

The review protocol was preregistered in the International Prospective Register of Systematic Reviews (PROSPERO 2023 CRD42023364807).

### Eligibility criteria

Title-abstract and full-text screening was conducted according to predefined eligibility criteria, which were developed according to the PICOS framework. The PICOS criteria, together with additional considerations not included in the PICOS framework, such as further definitions of the context, included publication types (randomized controlled trials (RCTs), quasiexperimental studies with at least one pretest and one posttest) or database filters used to specify the language and the publication date, are summarized in Table 1.

**Table 1:** Inclusion and exclusion criteria

Study characteristics	Inclusion criteria	Exclusion criteria
Population	nurses (assistants and registered nurses) working in inpatient care (e.g., hospitals, nursing homes)	a. nurses working in settings other than inpatient care settings b. interventions targeted at other healthcare workers
Intervention	organizational-interventions that were implemented to deal with pandemic-associated challenges in inpatient care and that shape the way nurses’ work is organized, designed and managed from a ‘top-down’ perspective (e.g., redesign of work tasks, activities, relationships, and responsibilities)	a. no intervention b. interventions not dealing with pandemic-associated challenges in inpatient care c. interventions only at the individual level (training, awareness raising/educational interventions)
Comparison	compared to baseline or to no/other intervention	no comparison
Context	at the organizational level of the workplace during the COVID-19 pandemic	a. outside the workplace b. outside of organizational level c. not related to the COVID-19 pandemic
Outcome		none
Study design	experimental/interventional studies: a. randomized controlled trial (RCT) b. quasiexperimental studies with at least one pre- and one posttest (e.g., controlled before and after studies with or without control group, interrupted time series, etc.)	a. observational studies (e.g., case‒control, cohort, cross-sectional, case-series) b. qualitative study designs (e.g., case studies) c. ecological studies d. proportional mortality ratio e. historically controlled studies
Publication details	publication time: between 01.01.2020 and 13.03.2023. languages: German or English-language primary studies published: a. in journal (peer reviewed) b. preregistration database	a. meta-analyses, reviews, editorials, letters to the editor, study protocols, commentaries

### Information sources and search strategy

Six databases including nursing and psychology literature were searched: PubMed, PsycINFO, PsycARTICLES, CINAHL, MedRxiv, and PsyArXiv. The latter two preprint databases were included to identify the most recent studies published during the COVID-19 pandemic. The search strategy consisted of keywords related to the population (e.g., assistants and registered nurses working in acute care hospitals or nursing homes), the pandemic context (e.g., COVID-19, SARS-CoV-2), the organizational intervention (e.g., intervention, training, work design) and the study design (e.g., RCT, quasiexperiment). Keywords related to outcomes were not specified because the review focused primarily on interventions delivered to manage a broad range of work stressors during the COVID-19 pandemic. The search was restricted to studies published in English and German, was performed on 13.03.2023 and covered all available studies published since 01.01.2020 (for the full search strategy, see Supplementary 3: Tables S1 and S2).

### Study selection process

A checklist with definitions of each PICOS element was prepared in advance and completed by two reviewers following a prespecified check sequence (see Supplementary 3: Figure S1 and Table S3). One reviewer screened and selected a total of 990 studies identified on the basis of their titles and abstracts. Together with a second reviewer, an independent decision on the inclusion or exclusion of a random set of 100 of the 990 identified publications was made to pilot the checklist and calculate interrater agreement. The program Rayyan [29] was used as a collaboration tool. The reviewers categorized title and abstracts for full-text screening as follows: ‘include’, ‘maybe’ or ‘exclude’. The interrater agreement was ‘moderate’ (*κ* = 0.55; 30). After discussing changes to the checklist, we reran the procedure with a new set of 100 random title and abstracts (only using ‘include’ and ‘exclude’ categories), resulting in ‘fair’ agreement (*κ* = 0.34; 30). The reason for the lower agreement may be that the reviewers conducted their screenings at different time points and had an additional work group meeting between those time points. Following this conventional double screening, the project team agreed on studies for further selection. After title-abstract screening, 141 full texts were screened by one reviewer, supplemented by spot checks performed by a second reviewer for 20% of the abstracts or full texts. In cases of disagreement, the entire research team was consulted for a final decision. After the study selection process, 12 studies were included in the review.

### Data extraction

Extraction tables were developed during the data extraction process and were spot-checked by a second reviewer. The custom-made data extraction form included an intervention description developed via the TIDieR checklist [28] and can be found in Supplementary 3: Table S4.

### Study risk of bias assessment

For the risk of bias assessment, we selected different validated tools depending on the study design, as recommended by Seidler et al. [31]. As recommended in the Cochrane Handbook for systematic reviews of interventions [32], we applied the Cochrane risk-of-bias tool for randomized trials – version 2 (RoB-2) tool [33] for randomized studies and the Risk Of Bias In Non-randomized Studies - of Interventions (ROBINS-I) tool [34] for nonrandomized studies. The risk of bias assessment considered confounding, randomization/selection of participants, classification of interventions, deviations from the intended intervention, missing outcome data, measurement of outcomes and selection of reported results. A risk of bias assessment was conducted for each study outcome and spot checked by a second reviewer (see Supplementary 3: Tables S5 and S6). Conflicts were discussed between the two reviewers. If no consensus could be reached, the whole project team discussed the issue.

### Effect measures

We did not conduct quantitative effect size synthesis. Whether an effect measure was reported or not was noted in the data extraction form. In the presentation of the results, only the significance and direction of relationships were tabulated (see Supplementary 3: Table S4).

### Evidence synthesis methods

We performed a synthesis without meta-analysis of the results and considered the risk of bias to answer the guiding questions (e.g., outcome, significance, and study quality; see Supplementary 3: Tables S8 and S9). The body of evidence for single outcomes (e.g., with the Grading of Recommendations, Assessment, Development and Evaluation (GRADE) approach, [35]) was not rated, as there were no studies on comparable interventions with the same outcome measure.

Since the comparability among the included interventions was limited, no quantitative synthesis was performed. Therefore, no meta-analysis of effect sizes was conducted, and no reporting bias was calculated.

## Results

### Study selection

The search yielded 990 records after the automatic elimination of duplicates. After screening the titles and abstracts of these records with the help of a prespecified check sequence of the selection criteria (see Supplementary 3: Figure S1 and Table S3), 158 records remained for full-text screening. By means of title-abstract screening, 15 records were pending study results. To ensure that of these 15 records, no more recent publications had been neglected, we performed additional manual searches in Google and Google Scholar on the basis of the information given by the title-abstract screening. Two full texts were found and retrieved for full-text screening but ultimately did not meet the inclusion criteria. The eligibility of a total of 141 retrieved full texts was assessed, resulting in 12 intervention studies (all published in peer-reviewed journals) that met the inclusion criteria. Even though the search included records from preprint databases, none of the records identified were included in the final sample.

There were studies that did not meet all of the inclusion criteria. First, the inclusion criteria for the inpatient nurse population, i.e., assistants and registered nurses working in acute care hospitals or nursing homes, were very strict. Some studies included patients, residents, or their caregivers as the primary target group with only the goal of improving the quality of care (e.g., [36–39]). These studies changed the work of nurses, for example, by activating a task force in the event of nursing home resident deaths [36]. However, these studies were excluded because the benefits or harms of the interventions for nurses were neither measured nor discussed. Other studies regarded healthcare workers as a whole group and did not distinguish their results between nurses and other staff (e.g., [40–44]). These studies were excluded, as no reliable nurse-specific statements could be derived regarding the intervention effects.

**Fig. 1:**
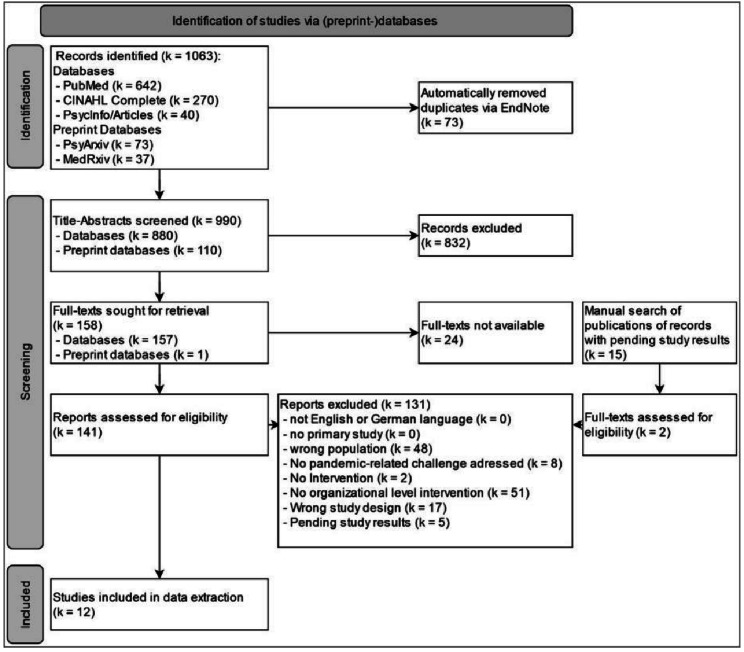
PRISMA flow-diagram [45]

Figure 1 shows the study selection process according to the Preferred Reporting Items for Systematic Reviews and Meta-Analyses (PRISMA) flow chart.

### Study characteristics

The study characteristics are presented in Table 2. All the included intervention studies were carried out in hospitals, four of which were specialized COVID-19 hospitals. The studies included samples from different units: intensive care units (k = 4; [46–49]), emergency departments (k = 2; [50, 51]) or the entire hospital (k = 5; [26, 52–55]). In one study, the hospital unit was not reported [56]. The mean sample size of the nurses was approximately 138 (range: 30 to 363).

**Table 2:** Study information, statistically significant and nonsignificant outcomes and overall risk of bias assessment

Intervention (brief name)	Country	Study population	Study design	Time frame, frequency/duration, intervention & prevention type	Statistical significance and direction of outcomes	Statistically nonsignificant outcomes	Overall risk of bias assessment
**Integrated workplace violence management intervention** (organizational component included regular team debriefings and feedback, Chang et al., [50])	Taiwan	Emergency nurses of COVID-19 hospital (90.7% female) IG *n* = 39 CG *n* = 36 (standard 1-hour in-service class only)	Cluster-randomized, pre- and posttest, controlled trial using parallel-groups	- conducted in 2020 12 sessions of at least 1 h - frequency/duration not reported - multilevel intervention - prevention secondary	Over group and time: ↑ goal commitment (*p* < .001) ↑ occupational coping self-efficacy (*p* < .001) ↑ confidence in managing violence (*p* < .001) ↑ attitudes toward aggressive behavior and explanation of violence (*p* < .001)	Attitudes toward aggression in Emergency Department	some^a^
**Professional development intervention simulation** (new or changed work activities in practicum, Goldsworthy et al., [46])	Canada	Critical care unit nurses (89.5% female); both groups from different hospitals IG *n* = 182 CG *n* = 181 overall dropout rate 61.99%	Quasiexperimental nonequivalent control group design with multiple points of measurement pre-post	- over the course of a one year period; 324-hr self-paced, critical care certificate program with Online Theory Component: 315 h (6 courses) and Simulations intervention: 39 h - ICU preceptored clinical practicum over ten 12 h-shifts (120 h) - multilevel intervention - secondary prevention	Group differences at (T3) controlled by (T0) measurements: ↑ intent to stay in the unit in IG compared to CG (*p* = .02) ↑ intent to stay in the profession in IG compared to CG (*p* < .001) mediator analysis for perceived organizational support ↑ direct effect: professional development intervention predicted higher intent to stay in the profession (*p* < .05) ↑ indirect effect: perceived organizational support mediated the relationship between professional development and the intent to stay in the profession (*p* < .05)	Group difference at (T3) in intent to stay in the organization	serious^b^
**Aromatherapy on ward** (Hung et al., [53])	Taiwan	Nursing staff (100% female) from different inpatient units (convenience sample) IG *n* = 30 13.33% dropout rate	Pre-posttest design	- for 4 weeks (during the second COVID-19 outbreak from April – June 2021) exposure to aroma diffused scent on ward twice every weekday (Friday-Monday) at 8:00–12:00 a.m. and 16:00–20:00 p.m. - job-oriented approach - secondary prevention	Objective measurements: statistically significant change in physical stress indicators only for subgroups, e.g.: ↑ ICU nurses’ physical indicators for a higher level of stress (activities of parasympathetic and sympathetic nervous system) after the intervention compared to before (*p* < .05) Subjective measurements ↓ nurse stress: Work concerns (*p* = .029) ↓ overall burnout score (= degree of fatigue; *p* = .017)	Physical stress indicators over all participants (heart rate variability) nurse stress questionnaire overall burnout score: client-related burnout	serious^b^
**Instrumental support and coaching leadership** (Kumar & Jin, [47])	Pakistan	COVID-19 frontline nurses of 107 government hospitals (41.7% female) working 12 h shifts (convenience sample) IG *n* = 319	Pre-posttest design	- started July 2020 - provision of resources was followed by a 3-month interval - job-oriented approach - primary and secondary prevention	↓ instrumental support decreases the undesirable effect of emotional labor on job stress (*p* < .001) ↓ coaching leadership decreases the undesirable effect of job stress on emotional exhaustion (*p* < .001)		moderate^b^
**Triggered palliative medicine consults** in the medical intensive care unit (Piscitello et al., [49])	USA	ICU nurses at one hospital nurses: IG *n* = 48 20% dropout rate patients: IG *n* = 50 CG *n* = 57	Pre-posttest design	- for 6 weeks (during the height of the second wave of the COVID-19 pandemic), continuous checks if patients met the criteria for triggered palliative medicine consults; consults must be seen within 24 h of ICU admission; criteria for family visits must be met by day 3 of admission and evaluated every 5–7 days - job-oriented approach - primary prevention	Primary nurse outcomes: ↓ nurse turnover intention due to moral distress (*p* = .006) secondary patient outcomes: ↓ rate of documented alternate decision makers (*p* < .001) ↓ discharge rate to facility or hospice (*p* < .001) ↓ time to transition to ‘do not resuscitate’ status (*p* = .029) ↓ days from ICU admission to palliative consult (*p* < .001) ↓ patient costs for specific subgroups lower than in control group (e.g., *p* = .003 for patients with do not resuscitate orders)	Primary nurse outcome: pre-post difference in moral distress secondary patient outcomes: overall costs per patient in the intervention group compared to CG; rate of do not resuscitate code status in the IG vs. CG; no decrease in the median ICU length of stay	moderate^b^
**Proactive organizational approach** (nurse environment, nurse staffing, workload, competence and learning motivation, participation, autonomy, process-focused unit-level intervention, healthcare surveillance, Zaghini et al., [26])	Italy	Frontline COVID-19 nurses (75.5% female) from a COVID-19 hospital (convenience sample) IG *n* = 350 8% dropout	Mixed methods one group pre-posttest design	- proactive planning started after ‘patient zero’ was identified with COVID-19 in Italy - 3 months of intervention from March 2020 to Mai 2020 (exponential increase in COVID-19 cases and lockdown in March) - multilevel intervention - primary and secondary prevention	Compared to baseline ↓ job-related stress (*p* < .001) ↑ job satisfaction (*p* < .001) ↑ quality of life (*p* = .003)	Single facets of job-related stress, satisfaction, and quality of life	serious^b^
**Holistic sleep improvement strategies** (scientific human resource management, comfortable sleep environment, self-relaxation/-adjustment, humanistic care, Y. Zhang et al., [55])	China (Wuhan)	Frontline COVID-19 nurses (96.2% female) from a COVID-19 hospital (convenience sample) IG *n* = 52	One group pre-posttest design	- conducted in February 2020 - the implemented strategies were practiced for 4 weeks - multilevel intervention - primary and secondary prevention	↑ Overall Sleep Quality Index compared to baseline (*p* = .004) sleep quality facets compared to baseline: ↑ subjective sleep quality (*p* = .016) ↑ sleep efficiency (*p* = .015) ↓ sleep disturbances (*p* = .007)	Sleep quality facets compared to baseline: sleep latency, sleep duration, sleep medication, daytime dysfunction	serious^b^
***Studies on rest break organization***							
**Motivational Messages Sent to Emergency Nurses** (short break triggered by messages, Goktas et al., [51])	Turkey	Emergency nurses from two designated pandemic hospitals (53.5% female) working only day shifts IG *n* = 33 CG *n* = 32 (no intervention) 7.6% dropout rate	Randomized-controlled experimental study	- over a duration of 21 days (July August 2021), nurses received three motivational messages per day and would take 5–10 min breaks to check their phones - multilevel intervention - secondary prevention	Over group and time: ↑ job satisfaction (*p* < .05) ↑ communication skills (*p* < .05) ↓ lower compassion fatigue (*p* < .05)		some^a^
**Resilience bundle for emergency nurses** (‘serenity room’, structured debriefing, relaxation and mindfulness, Haugland et al., [52])	USA	Emergency nurses (89.6% female) of level I trauma center with > 30% of COVID-19 patients IG *n* = 47 loss-to-follow rate of 51.06%	Mixed-method pre-posttest design	- conducted in 2021 - 15 weeks with an implemented daily practiced resilience bundle - multilevel intervention - secondary prevention	↑ self-reported resilience (T1) compared to baseline (*p* = .003)	Perceived stress score self-reported resilience (T2) compared to baseline	critical^b^
**Healing Touch intervention** during additional breaks (Rosamond et al., [54])	USA	Inpatient nurses (93% female) of various units (acute care, critical care, perioperative care, inpatient dialysis care) from different hospitals IG *n* = 75 CG *n* = 75 (deep breathing group)	Mixed-method cluster randomized controlled trial with matched pairs randomization	- conducted in February 2020 - during 12-hr day work shift within a 15 min work break (4–7 min Healing Touch session) - job-oriented approach - secondary prevention	Subjective measurements ↓ stress symptoms post treatment (T1; *p* < .001) and follow-up (T2; *p* = .014) compared to control group objective measurements ↓ respiratory rate at follow-up (T2; *p* < .001) compared to the control group	(T1 and T2) heart rate, systolic blood pressure (T1) respiratory rate	high^a^
**Use of ‘serenity lounges**’ (Pagador et al., [56])	USA	registered nurses (84.6% female) across 10 inpatient units of one medical center IG *n* = 67 22.39% dropout	Pre-posttest design	- since November 2020 (evaluation is ongoing) - daily access to serenity lounges over 7 months (included a spike in the number of admitted COVID-19 patients between December 2020 and February 2021) - job-oriented approach - secondary prevention	Use of serenity lounge: ↓ less feelings of emotional exhaustion, burnout, frustration, being worn out, stress, anxiety (*p* < .001) compared to before use duration of massage chair use: ↓ for 10–20 min than < 10 min: higher reduction in feeling worn out (*p* = .03), emotional exhaustion (*p =* .04), and anxiety (*p =* .01) compared to before for < 20 min than < 10 min: higher reduction in anxiety (*p* = .03) compared to before	no sign. higher reduction in emotional exhaustion, burnout, frustration, being worn out, stress, after > 20 min of use	critical^b^
**Virtual reality relaxation (**Nijland et al., [48])	Nether-lands	ICU nurses (85% female) working with COVID-19 patients at one hospital IG *n* = 86 23.26% dropout rate	Pre-posttest design	- over a duration of 3 months (May-June 2020, during the first COVID-19 wave) 24 h access to a separate room during work shifts on COVID-19 wards; with recommended time of use of at least 10 min - job-oriented approach - secondary prevention	↓ less perceived stress (*p* < .005) immediately after the intervention compared to before		serious^b^

Note. IG = intervention group, CG = control group; ↑ = increased, ↓ = decreased; the p value indicates a statistically significant difference (e.g., between the IG and CG/before and after the intervention). In line with the APA 7th Edition guidelines, the exact p value is depicted (if given by the included study), unless p was < 0.001; T refers to the time point of measurement (T0 = baseline before intervention, T1 = next measurement, etc.)

^a^ risk of bias (RoB-2, 33) = low / some / high concerns

^b^ risk of bias (ROBINS-I, 34) = low / moderate / serious / critical

Organizational interventions were conducted in eight different countries: the USA (k = 4), Taiwan (k = 2), and Canada, China, Italy, the Netherlands, Pakistan, and Turkey (k = 1 for each).

The first reported interventions were carried out in February 2020 (k = 3). Some studies (k = 5) indicated that the intervention was implemented during a COVID-19 surge. The median intervention interval was three months, with the longest intervention interval lasting up to one year (i.e., a learning curriculum was evaluated that included the implementation of new or changed work tasks for nurses). The shortest intervention interval was one 15-minute break (healing touch session) during a 12-hour shift.

Seven studies implemented more than one single intervention. The highest number of interventions was implemented in a study of ‘holistic sleep improvement strategies’, with 13 interventions [55]. Half of the included studies (k = 6) included multilevel interventions that combined a person-oriented approach and a job-oriented approach.

All included studies examined nurse-related outcomes and used at least one subjective outcome assessment. Only one study [49] considered patient (e.g., discharge rate to non-intensive-care units) and hospital outcomes (e.g., patient costs) in addition to nurse-related outcomes (turnover intention).

### Risk of bias assessment

We assessed the risk of bias to estimate whether an intervention effect in a study was due to an effective intervention or other (confounding) factors. The overall result of each study assessment are shown in Table 2 (for full report see Supplementary 3: Tables S5 and S6).

The most common methodological problems that increased the risk of bias were, for example, convenience sampling (k = 12), the lack of a study protocol or preregistration (k = 9), confounding due to the lack of a control group (k = 7), unclear hypotheses (k = 6), no assessment of intervention adherence or implementation success (k = 3), and a high dropout rate (k = 3). Furthermore, in many studies, confounding variables, such as a high workload during the pandemic (k = 6), could have had an unmeasured and uncontrolled influence on the use of the intervention, the completion of surveys, adherence, and dropout and thereby on the measured effect of the intervention.

In general, according to the ROBINS-I tool (k = 9), two studies were rated as critical, five studies were rated as having a serious risk of bias, and two studies were rated as having a moderate risk of bias. No study was rated as having a low risk of bias. The results of the RoB-2 tool (k = 3) revealed one study with a high risk of bias and two studies with some risk of bias (see Table 2).

### Synthesis of results

In view of the seven research questions, a synthesis without meta-analysis of the results was performed. A short overview is provided in Table 2. For a detailed description according to the TIDieR checklist [28], see Supplementary 3: Tables S4, S7, S8 and S9.

#### Circumstances and rationale: ‘Why?’ (Research Question 1; RQ 1)

Notably, in no study were working conditions assessed before the intervention was implemented. Almost all (k = 11) of the included studies used literature reviews that supported the rationale for the intervention. Only one study mentioned unit-specific problems as the rationale for (and a hindrance to) the intervention [52].

RQ 1: The most frequently given rationale for the intervention was the high workload of nurses (k = 5; [26, 49, 51, 53, 55]). Other reported work stressors were insufficient staffing/a low nurse-to-patient ratio (k = 3; [26, 46, 52]) and limited medical resources (k = 2; [51, 53]). The work stressors addressed were allocated across the five components of the work system (activities, resources, the physical environment, and temporal or social aspects). Studies have identified the need for action with respect to all components of the work system.

For some studies (k = 3; [48, 54, 56]), the intervention rationale was not based on work stressors but rather on strain experienced by nurses or negative consequences for hospitals and patients. This reflects the secondary preventive approach to create structures to buffer pandemic-associated work strain or adverse consequences (see RQ 3).

A full description of the rationale for each study can be found in Supplementary 3: Table S7.

#### Intervention description: ‘What was provided by whom in what way?’ (RQs 2–5)

RQ 2: In general, the studies applied very different interventions in terms of their rationales, measures, and outcomes. The organizational interventions directly (re)designed work aspects in a ‘top down’ manner for all five components of the work system.

RQ 3: The prevention approach could be derived from the work stressors extracted for RQ 1 and whether these work stressors were modified directly (primary prevention) or other aspects of the work were changed to buffer negative effects (secondary prevention).

A frequent approach among secondary prevention interventions (k = 8) was to change the organization of nurses’ rest breaks (k = 5). For example, Goktas et al. [51] reported the excessive workload of emergency nurses as the rationale for their study. Instead of modifying the workload itself, their intervention aimed to improve motivation through smartphone messages. Emergency nurses were allowed to take short breaks to check their phones for these messages.

In general, all five studies did not discuss any theoretical rationale on why and how (re)organizing work breaks could ameliorate the negative effects of work stressors. The design of rest breaks involved changes to different work system components (time, the physical environment, resources, and activities).

An exclusively primary preventive approach was chosen by one theory-based study [49] that combined subjective and objective measurements and had one of the lowest risks of bias (moderate ratings) among the quasiexperimental studies. Piscitello et al. [49] reasoned that the moral distress of nurses likely increased during the COVID-19 pandemic. To provide sufficient care for critically ill patients, palliative medicine consultations were conducted, and family meetings were arranged.

Other studies (k = 3) combined primary and secondary intervention approaches [26, 47, 55]. These studies modified the work stressors themselves and (re)organized other aspects of the work system to buffer their effects. For example, the theory-based intervention study by Kumar and Jin [47] was one of two qualitatively well-rated quasiexperimental studies. The purpose was to decrease associated infection risks and high emotional demands due to the lack of PPE. Consequently, nurses were provided with instrumental support (for example, the provision of protective equipment) as a primary prevention approach. It was hypothesized that perceived instrumental support would mitigate the adverse effects of emotional labor on work stress in an emergency. The coaching leadership style, as a secondary prevention approach, was hypothesized to reduce the adverse effects of work stress on emotional exhaustion. However, the study revealed that perceived instrumental support itself could not fully weaken the negative effect of emotional labor on work stress. Positive feedback and clear communication with staff and social support provided via a coaching leadership style were found to be protective factors against work stress.

RQ 4: The organizational interventions modified aspects of the five components of the work system. Often, more than one work system component was changed by intervention (k = 9; [26, 47, 48, 50–52, 54–56]). Note that the studies did not further assess the actual changes in the work system.

The component most often considered was work activities (k = 10; [26, 46–52, 54, 55]). This very diverse category included several themes, e.g., new or changed work activities after training sessions, triggers for certain work activities, restructuring work processes, participation, or the implementation of break activities.

The second most frequently modified component was (human) resources for nurses (k = 7; [26, 47, 48, 50, 52, 55, 56]). An example is the proactive approach of Zaghini et al. [26]. In their study, an online tool was developed to provide transparent information on COVID-19-related procedures (e.g., reporting guidelines and updated information by the national authority).

The least modified component of the work system was the social aspect (k = 3; [26, 47, 55]). The studies implemented the coaching leadership style [47], positive feedback and acknowledgment [55], and opportunities for discussion regarding care adjustments, as well as participation [26].

The approach to (re)organize rest breaks was described above (k = 5). Changes in the organization of rest breaks were also distributed across different components of the work system. The five studies reorganized rest breaks related to the time component (e.g., short breaks; k = 3; [48, 51, 54]), the physical environment (e.g., break rooms; k = 3; [48, 52, 56]), the activities during breaks (e.g., going for a walk; k = 2; [48, 54]) and the component resources (e.g., massage chairs; k = 3; [48, 52, 56]).

However, the risk of bias ratings raised concerns about confounding and participant selection (see Supplementary 3: Tables S5, S6, S8 and S9).

RQ 5: Six studies applied a multilevel intervention approach combining job-oriented and person-oriented measures. In two studies, organizational interventions served only to formalize and promote newly learned behavior at the individual level via the person-oriented approach [46, 50]. In other studies, multilevel interventions were designed directly in a holistic manner [26, 52, 55].

#### Outcomes and ‘how well’ did the intervention work? (RQ 6–7)

An overview of the outcomes can be found in Table 2. For a detailed presentation of the outcomes considered and an evaluation of the strengths and weaknesses, see Supplementary 3: Tables S8 and S9.

With respect to RQ 6, all studies addressed nurse outcomes. The nurse outcomes included (job) stress- and resilience-related outcomes (k = 6), turnover intention (k = 2), job satisfaction (k = 2), and other outcomes. Only one study [49] additionally assessed patient (e.g., patient discharge rates) and hospital outcomes (patient costs). The body of evidence for the mentioned outcomes was not rated and should be considered very low due to methodological limitations and heterogeneity of the interventions among studies. That is, for each outcome category, the corresponding interventions differed.

With respect to RQ 7, we found that the discussion of factors that could promote or hinder the intervention was rather limited in the studies (k = 7; [26, 46, 47, 50, 51, 53, 54]). COVID-19-related challenges, such as visitor restrictions that hindered communication with patients’ families [49], high workloads that did not allow for breaks [48], and staff turnover [52], have been reported. With respect to the studies targeting rest break organization, workload and understaffing [48] and high absence due to sickness and turnover rates [52] presented a common hindrance. Only Pagador et al. [56] discussed the factors that (in their opinion) promoted the implementation of their ‘serenity lounge’ intervention during a COVID-19 wave. First, they were able to maintain a nurse‒patient ratio of 1:4 or 1:6 (depending on shifts). Second, the authors reported on the nursing team cohesion, leadership support, and early participation and commitment of the head nurses as factors promoting the intervention. A detailed description of the characteristics and hindrances of the interventions is presented in Supplementary 3: Table S4.

## Discussion

This preregistered systematic review included 12 intervention studies (published between January 2020 and March 2023) conducted during the COVID-19 pandemic with nurses from eight different countries. We updated the findings of Nicolakakis et al. [21], and our results support some of the recommendations for healthcare workers (e.g., regarding organizational support) that were derived on the basis of earlier pandemics [17].

Nicolakakis et al. [21] synthesized evidence on organizational interventions to protect the mental health of healthcare workers (mainly nursing professionals) during epidemics and pandemics. They identified five observational studies that were conducted during the COVID-19 pandemic. However, Nicolakakis et al. [21] reported low to very low confidence in intervention effectiveness. The present review also considered intervention studies with mental health outcomes, but we additionally confined our scope to prospectively planned intervention studies with at least one premeasurement and one postmeasurement.

### Multilevel interventions

Most of the studies related to COVID-19 (k = 3) included in the review of Nicolakakis et al. [21] were multilevel interventions that combined job-oriented and person-oriented approaches to improve the mental health of healthcare workers, similar to the ‘resilience bundle’ [52], ‘holistic sleep improvement strategies’ [55], and ‘proactive organizational approach’ [26] included in the present review.

The combination of both approaches is a common successful concept in occupational health research [57, 58]. For example, a recent systematic review of studies of organizational interventions revealed that multilevel interventions for employees resulted in better and longer-lasting burnout prevention than did pure single-level interventions [59].

When both approaches are used, individuals (e.g., nurses) can influence their experiences and emotions by adapting and shaping their behavior to situations (e.g., changes throughout the COVID-19 pandemic; [60]). Additionally, the job-oriented approach can influence individual behavior and improve an individual’s ability to cope with work stressors. The mutual influence of organizational interventions and individual-level interventions has also been highlighted in the work design literature regarding management research and occupational health psychology [61].

In summary, performing multilevel interventions during crisis situations and pandemics could promote the health of nurses and health care management in hospitals (e.g., staff, patients) because such interventions comprehensively address multiple different and interacting work stressors in such a dynamic sociotechnical work system [19]. However, in the present review, the quality of the three mentioned studies was limited, which was also discussed by Nicolakakis et al. [21].

### Organizational interventions to improve organizational support

The study by Kumar and Jin [47] examined the effect of organizational support by providing instrumental and psychological resources. Organizational support is defined as nurses’ perceptions that their organizations value their contributions and care about their well-being [62]. Healthcare organizations could support healthcare workers instrumentally and emotionally by providing access to PPE, clear communication with staff, psychological support and sufficient time and opportunities (e.g., rooms) for rest breaks [17, 63, 64], which should be beneficial, particularly in times of organizational change [65].

Kumar and Jin [47] reported that access to PPE is more common than infection control since it may influence nurses’ work stress. In particular, work stress mediated the adverse influence of emotional labor on emotional exhaustion before the provision of PPE (instrumental support) and only partially after. When PPE was provided to frontline nurses, the stress-promoting effect of emotional labor was significantly weaker. However, the scarcity of medical resources is a challenge during a global crisis [5]. For hospitals, the question is probably not whether PPE and other medical resources are effective but how to acquire these resources. In addition, nurse managers or head nurses can promote organizational support by providing emotional support, for example, through the use of a coaching leadership style [47]. In the review by Kisely et al. [17], a perceived lack of organizational support was considered a risk factor for adverse psychological outcomes in healthcare workers. Additionally, the protective factors included positive feedback, clear communication with staff, and social support. These factors were considered in the study by Kumar and Jin [47], supporting the buffering role of the coaching leadership style on the adverse effect of work stress on emotional exhaustion in nurses.

Adequate rest, short breaks, time off, and appropriate work shifts were also recommended by Kisely et al. [17] to prevent adverse psychological outcomes in the face of high job demands during epidemics. Aspects of rest breaks were reorganized in several studies of this review. High effort can lead to the depletion of an individual’s resources [66]. To regulate their effort expenditure, individuals need to recover their resources, for example, by taking temporary breaks from work [67]. Rest breaks can buffer the effect of high job demands (for example, high work intensity) on short-term strain (for example, fatigue) and therefore on long-term consequences (e.g., burnout; [68]). Different aspects of rest break organization, such as rest break activities and high-quality rest break areas, have been associated with better physical and psychological well-being among nurses outside the pandemic context [68]. However, future studies need to gather data on evidence-based recommendations to determine whether such recovery approaches also help nurses cope with high demands in a pandemic context.

In summary, the provision of PPE and other medical resources required during a pandemic addresses the lack of resources directly but also functions as organizational support. Adapting organizational support to the needs of nurses is important for creating tangible benefits and resources for their efforts [69]. Leadership strategies could increase feelings of organizational support [47]. With respect to the (re)organization of rest breaks, rest breaks could function as job resources to support nurses’ ability to cope if sources of work strain (e.g., work stressors such as high workloads) cannot be directly addressed. In addition, adequate staffing is needed to allow the use of rest breaks [56]. This was problematic even before the pandemic, when nurses already lacked opportunities for rest breaks [70].

#### Limited opportunities for primary interventions

We found that two-thirds of the included studies did not change work stressors themselves but rather other job-oriented aspects of work to support nurses.

However, as an example, the European Directive on measures to encourage improvements in the safety and health of workers at work (Directive 89/391/EEC) states that the source of risks must be addressed and that other interventions must be preferred over individual protective interventions [71].

This premise is even more important in the context of this review, where the antecedents of nurses’ stress also hindered them from using interventions such as rest breaks. Therefore, the question arises as to why the focus of the organizational interventions was on secondary prevention.

Organizations had to function under stressful time constraints and deal with the novelty of the pandemic, unclear or shifting goals, and ill-structured situations [72], which may have hindered the capacity of hospitals to address sources of work strain. For example, checklists for hospital disaster preparedness emphasize the importance of an immediate response to sudden high care demands to maintain system function [73]. Designing adequate human-centered work interventions [74] might be a secondary priority. However, according to Schmucker [70], high workloads and a lack of time were ongoing problems in professional nursing even before the pandemic.

These problems cannot be solved without increasing the number of personnel to meet the (growing) care demands [70]. In a global pandemic with a sudden rise in demand for care [6], the spontaneous acquisition of more personnel was even more difficult. An approach to this problem was to increase the expertise of existing personnel instead of the number of personnel [75] by retraining nurses in critical care. The only included study that examined these trainings was Goldsworthy [46], which implemented critical care training for nurses followed by the application of newly learned skills and work tasks. Furthermore, rehiring part-time working nurses could have been difficult during the COVID-19 pandemic. In a national German survey, more than one-third of the 8007 nurses who left their profession or worked part time stated that their readiness to reenter the job or switch from part-time to full-time employment decreased during the COVID-19 pandemic [76].

These arguments highlight the need to take action outside pandemics, not only to address the ongoing lack of personnel but also to prepare for future pandemics.

### Implications for practice

In general, it is important to address ongoing structural problems to retain and gain personnel to meet the growing demand for care. Improving the working conditions of nurses considering a human-centered work design [77] through organizational interventions could be one way to improve the attractiveness of the nursing position and therefore lead to increased recruitment [70]. The following recommendations can be made.

First, a participatory approach should be considered in the process of developing organizational interventions. The participation of nurses is an important predictor of successful organizational interventions [56, 78]. Organizations can facilitate the participation of their employees in interventions by involving them in initial discussions in the preparation phase or allowing them to identify key issues through surveys or focus groups in the screening phase. Teams of employees and managers can develop interventions in the action planning phase, and employees take active roles in the implementation phase. In the evaluation phase, employees can be involved in feedback sessions or follow-up surveys [79]. Zaghini et al. [26] promoted employee participation through continuous clinical and organizational audits, lectures, and workshops, which provided opportunities to discuss care adjustments.

Second, the complexity of the crisis must be acknowledged, as it affects the whole work system of nurses. Therefore, the best approach, at least theoretically, might be combining individual-level interventions (e.g., providing mindfulness training) with job-based interventions (e.g., making scheduling changes or designing work breaks).

Third, the literature and findings of this review can be used to anticipate pandemic-associated challenges for nurses in the future. One way to prepare for pandemics is by completing extensive checklists addressing hospital crisis preparedness [73]. These checklists consider issues such as logistics, human resources, triage, and communication, which are essential to keep care systems functioning [73]. Another step would be for hospitals to stay up-to-date with research. New evidence regarding the COVID-19 pandemic is still being published. On another note, collaborating with external consultants and researchers with expertise in work and organizational psychology may benefit hospital practice as well as further research [78].

### Implications for research

The main research question of this review was ‘What organizational interventions were provided?’ and not ‘How effective were the interventions?’. Therefore, future research should focus on the question of intervention effectiveness.

The findings of the summarized organizational interventions need to be replicated in similar contexts to determine whether these interventions are truly effective. Future studies should consider quality improvements in their study designs, for example, by controlling for confounding factors (e.g., nurse workload).

Furthermore, the present review, as well as the review by Nicolakakis et al. [21], identified problems with the reporting of interventions in the studies. To allow replication, interventions need to be described in more detail. Checklists such as the TIDieR checklist [28] are helpful in considering such details.

Furthermore, future research needs to evaluate the interventions considered herein in other contexts since the generalizability of the study findings is limited by the use of convenience sampling. The pandemic context may differ from country to country, from hospital to hospital, or even from unit to unit. There may also be cultural and legislative differences (for example, rest break culture and mandatory breaks) that must be considered when interventions are implemented.

This systematic review identified the most prominent research gap as evidence-based interventions for nurses working in nursing homes, as no study conducted in nursing homes met the strict criteria for inclusion in this review. Future research should focus on supporting scientific evidence of organizational interventions for nurses working in nursing homes to manage pandemic-related work stressors such as additional work activities (e.g., isolation protocols, pandemic-related protocols after death, establishment of contact with residents’ families) and social aspects (e.g., challenging behavior during pandemics; [80]).

### Limitations

One limitation is that our review probably included a small excerpt of the studies conducted. The pandemic context could have led to the cancellation of studies due to the high absence rates due to illness. In some cases, the pandemic context may have allowed for only retrospective studies that were not included in this review.

Second, the selection process was very strict. This led to the exclusion of all existing studies conducted in nursing homes. For example, we excluded a study in which a task force was formed to implement measures such as infection control and nurse support actions in the event of nursing home resident deaths [36].

Additionally, it is debatable whether the limitation to one population group was reasonable in light of a pandemic that affected the whole health care organization. For example, organizational interventions, such as managing whole infection pathways of visitors in hospitals, could reduce nurses’ fear of infection. However, if outcomes for nurses were not analyzed, such studies were excluded.

## Conclusions

The end of the COVID-19 pandemic was declared by the World Health Organization in May 2023 [81]. The pandemic had the consequences of global illness, death and exhaustion for patients, families, nurses and hospitals. There are several lessons to be learned from the COVID-19 pandemic.

In this context, the present systematic review adds to a small body of research aimed at improving the working conditions of nurses during the COVID-19 pandemic and provides a basis for future research on organizational interventions during pandemics to draw conclusions about their effectiveness. This review updated the findings of Nicolakakis et al. [21] by taking a wider view (more than just mental health outcomes) and by searching for non-peer-reviewed literature. We found that the use of human-centered work designs in organizational interventions can be pursued even during a pandemic crisis. Work stressors (such as a lack of PPE) should preferably be addressed directly, which can be difficult during a crisis. The promotion of adequate work breaks could be useful if the work stressors associated with strain and negative consequences cannot be changed directly. However, the same stressors (e.g., high workload) can hinder nurses from participating in offered intervention. This emphasizes the importance of directly addressing work stressors. Organizations can also support nurses by being sensitive to their needs (i.e., promoting the participation of nurses in different evaluation phases through surveys, active roles, and group sessions) and providing tangible benefits and resources (i.e., psychological support through leadership style and sufficient PPE).

## Electronic supplementary material

Below is the link to the electronic supplementary material.

Supplementary Material 1: Zink et al_PRISMA-Checklist (2020).

Supplementary Material 2: Zink et al_PRISMA DTA for Abstract Checklist.

Supplementary Material 3: Zink et al._Supplementary material_research data.

## Data Availability

The datasets supporting the conclusions of this article are included within the article and its additional files.

## Abbreviations

COVID-19: Coronavirus disease 2019
k or n: Study number or sample size
PPE: Personal protective equipment
PRISMA: *The Preferred Reporting Items for Systematic reviews and Meta-Analyses*
RoB: Risk of Bias
ROBINS: Risk Of Bias In Nonrandomized Studies of Interventions
RQ: Research question
SEIPS 2.0: Systems Engineering Initiative for Patient Safety, second version
TIDieR: Template for intervention description and replication

## References

[1] Dichter M, Kocks A, Meyer G, Stephan A. Pflege ist systemrelevant - Nicht nur in Corona-Zeiten: Gemeinsame Stellungnahme zum Internationalen Jahr der Pflegenden und Hebammen vor dem Hintergrund der Corona -Pandemie in Deutschland. [Nursing is systemically relevant - not only in corona times: Joint statement on the International Year of Nurses and Midwives against the background of the corona pandemic in Germany].

[2] Schulze S, Holmberg C. Bedeutung Und Belastung Von Pflegekräften während Der Corona-Krise. [Importance and strain on carers during the coronavirus crisis]. Public Health Forum. 2021;29:32–5. 10.1515/pubhef-2020-0114.

[3] Reuter M, Rigó M, Formazin M, Liebers F, Latza U, Castell S, et al. Occupation and SARS-CoV-2 infection risk among 108 960 workers during the first pandemic wave in Germany. Scand J Work Environ Health. 2022;48:446–56. 10.5271/sjweh.4037. 35670286 10.5271/sjweh.4037 PMC9888438

[4] Nguyen LH, Drew DA, Graham MS, Joshi AD, Guo C-G, Ma W, et al. Risk of COVID-19 among front-line health-care workers and the general community: a prospective cohort study. Lancet Public Health. 2020;5:e475–83. 10.1016/S2468-2667(20)30164-X. 32745512 10.1016/S2468-2667(20)30164-X PMC7491202

[5] Danesh MK, Garosi E, Golmohamadpour H. The COVID-19 pandemic and nursing challenges: a review of the early literature. WOR. 2021;69:23–36. 10.3233/WOR-213458. 10.3233/WOR-213458 34024803

[6] Xu H, Stjernswärd S, Glasdam S. Psychosocial experiences of frontline nurses working in hospital-based settings during the COVID-19 pandemic - A qualitative systematic review. Int J Nurs Stud Adv. 2021;3:100037. 10.1016/j.ijnsa.2021.100037. 34308373 10.1016/j.ijnsa.2021.100037 PMC8285218

[7] Falatah R. The impact of the Coronavirus Disease (COVID-19) pandemic on nurses’ turnover intention: an integrative review. Nurs Rep. 2021;11:787–810. 10.3390/nursrep11040075. 34968269 10.3390/nursrep11040075 PMC8715458

[8] Garcia C, Abreu L, Ramos J, Castro C, Smiderle F, Santos J, Bezerra I. Influence of Burnout on Patient Safety: systematic review and Meta-analysis. Medicina. 2019;55:553. 10.3390/medicina55090553. 31480365 10.3390/medicina55090553 PMC6780563

[9] Ślusarska B, Nowicki GJ, Niedorys-Karczmarczyk B, Chrzan-Rodak A. Prevalence of depression and anxiety in nurses during the First Eleven months of the COVID-19 pandemic: a systematic review and Meta-analysis. IJERPH. 2022;19:1154. 10.3390/ijerph19031154. 35162183 10.3390/ijerph19031154 PMC8834441

[10] Varghese A, George G, Kondaguli SV, Naser AY, Khakha DC, Chatterji R. Decline in the mental health of nurses across the globe during COVID-19: a systematic review and meta-analysis. J Glob Health. 2021;11:5009. 10.7189/jogh.11.05009. 10.7189/jogh.11.05009 PMC8053406 33884193

[11] Muller AE, Hafstad EV, Himmels JPW, Smedslund G, Flottorp S, Stensland SØ, et al. The mental health impact of the covid-19 pandemic on healthcare workers, and interventions to help them: a rapid systematic review. Psychiatry Res. 2020;293:113441. 10.1016/j.psychres.2020.113441. 32898840 10.1016/j.psychres.2020.113441 PMC7462563

[12] Park J-H, Jung S-E, Ha D-J, Lee B, Kim M-S, Sim K-L, et al. The effectiveness of e-healthcare interventions for mental health of nurses. Medicine. 2022;101:e29125. 10.1097/MD.0000000000029125. 35758346 10.1097/MD.0000000000029125 PMC9276241

[13] Velana M, Rinkenauer G. Individual-level interventions for decreasing job-related stress and enhancing coping strategies among nurses: a systematic review. Front Psychol. 2021. 10.3389/fpsyg.2021.708696. 34349711 10.3389/fpsyg.2021.708696 PMC8326445

[14] Lamontagne AD, Keegel T, Louie AM, Ostry A, Landsbergis PA. A systematic review of the job-stress intervention evaluation literature, 1990–2005. Int J Occup Environ Health. 2007;13:268–80. 10.1179/oeh.2007.13.3.268. 17915541 10.1179/oeh.2007.13.3.268

[15] International Labour Organization. Report of the Joint ILO/WHO Committee on Occupational Health (twelfth session, Geneva, 5–7 April 1995); 1995.

[16] Montano D, Hoven H, Siegrist J. Effects of organisational-level interventions at work on employees’ health: a systematic review. BMC Public Health. 2014. 10.1186/1471-2458-14-135. 24507447 10.1186/1471-2458-14-135 PMC3929163

[17] Kisely S, Warren N, McMahon L, Dalais C, Henry I, Siskind D. Occurrence, prevention, and management of the psychological effects of emerging virus outbreaks on healthcare workers: rapid review and meta-analysis. BMJ. 2020;369:m1642. 10.1136/bmj.m1642. 32371466 10.1136/bmj.m1642 PMC7199468

[18] Carayon P, Bass EJ, Bellandi T, Gurses AP, Hallbeck MS, Mollo V. Sociotechnical systems analysis in health care: a research agenda. IIE Trans Healthc Syst Eng. 2011;1:145–60. 10.1080/19488300.2011.619158. 22611480 10.1080/19488300.2011.619158 PMC3351758

[19] Holden RJ, Carayon P, Gurses AP, Hoonakker P, Hundt AS, Ozok AA, Rivera-Rodriguez AJ. SEIPS 2.0: a human factors framework for studying and improving the work of healthcare professionals and patients. Ergonomics. 2013;56:1669–86. 10.1080/00140139.2013.838643. 24088063 10.1080/00140139.2013.838643 PMC3835697

[20] Bethel C, Reed PG, Brewer BB, Rainbow JG. Selecting a theoretical framework to guide research on the COVID-19 pandemic impacts on nursing care delivery and the critical care work system (using Reed’s Intermodern approach to theory critique). Appl Nurs Res. 2022;63:151513. 10.1016/j.apnr.2021.151513. 35034706 10.1016/j.apnr.2021.151513 PMC8494499

[21] Nicolakakis N, Lafantaisie M, Letellier M-C, Biron C, Vézina M, Jauvin N et al. Are Organizational Interventions Effective in Protecting Healthcare Worker Mental Health during Epidemics/Pandemics? A Systematic Literature Review. IJERPH. 2022. 10.3390/ijerph19159653 10.3390/ijerph19159653 PMC9368524 35955009

[22] Beneria A, Arnedo M, Contreras S, Pérez-Carrasco M, Garcia-Ruiz I, Rodríguez-Carballeira M, et al. Impact of simulation-based teamwork training on COVID-19 distress in healthcare professionals. BMC Med Educ. 2020;20:515. 10.1186/s12909-020-02427-4. 33349248 10.1186/s12909-020-02427-4 PMC7751744

[23] Blake H, Yildirim M, Wood B, Knowles S, Mancini H, Coyne E, Cooper J. COVID-Well: evaluation of the implementation of supported Wellbeing centres for Hospital employees during the COVID-19 pandemic. Int J Environ Res Public Health. 2020. 10.3390/ijerph17249401. 33333913 10.3390/ijerph17249401 PMC7768437

[24] Giordano F, Cipolla A, Ungar M. Building resilience for healthcare professionals working in an Italian red zone during the COVID-19 outbreak: a pilot study. Stress Health. 2022;38:234–48. 10.1002/smi.3085. 34312986 10.1002/smi.3085 PMC9292917

[25] Zhu Z, Xu S, Wang H, Liu Z, Wu J, Li G, et al. COVID-19 in Wuhan: sociodemographic characteristics and hospital support measures associated with the immediate psychological impact on healthcare workers. eClinicalMedicine. 2020;24:100443. 10.1016/j.eclinm.2020.100443. 32766545 10.1016/j.eclinm.2020.100443 PMC7311903

[26] Zaghini F, Fiorini J, Livigni L, Carrabs G, Sili A. A mixed methods study of an organization’s approach to the COVID-19 health care crisis. Nurs Outlook. 2021;69:793–804. 10.1016/j.outlook.2021.05.008. 34176670 10.1016/j.outlook.2021.05.008 PMC8114768

[27] Nielsen KM, Noblet A. Organizational interventions: where we are, where we go from here? In: Nielsen KM, Noblet A, editors. Organizational Interventions for Health and Well-being: A Handbook for Evidence-Based Practice. pp. 1–22.

[28] Hoffmann TC, Glasziou PP, Boutron I, Milne R, Perera R, Moher D, et al. Better reporting of interventions: template for intervention description and replication (TIDieR) checklist and guide. BMJ. 2014;348:1–12. 10.1136/bmj.g1687. 10.1136/bmj.g1687 24609605

[29] Ouzzani M, Hammady H, Fedorowicz Z, Elmagarmid A. Rayyan—a web and mobile app for systematic reviews. Syst Rev. 2016. 10.1186/s13643-016-0384-4. 27919275 10.1186/s13643-016-0384-4 PMC5139140

[30] Landis JR, Koch GG. The measurement of Observer Agreement for Categorical Data. Biometrics. 1977;33:159. 10.2307/2529310. 843571

[31] Seidler A, Nußbaumer-Streit B, Apfelbacher C, Zeeb H. Rapid Reviews in Zeiten Von COVID-19 – Erfahrungen Im Zuge Des Kompetenznetzes Public Health zu COVID-19 und Vorschlag eines standardisierten vorgehens. [Rapid Reviews in the Time of COVID-19 - experiences of the Competence Network Public Health COVID-19 and proposal for a standardized Procedure]. Gesundheitswesen. 2021;83:173–9. 10.1055/a-1380-0926. 33634462 10.1055/a-1380-0926 PMC8043586

[32] Boutron I, Page MJ, Higgins JPT, Altman DG, Lundh A, Hróbjartsson A. Considering bias and conflicts of interest among the included studies. In: Higgins J, Thomas J, Chandler J, Cumpston M, Li T, Page MJ, Welch VA, editors. Cochrane handbook for systematic reviews of interventions. Hoboken, NJ, Chichester: Wiley Blackwell; 2019. pp. 177–204. 10.1002/9781119536604.ch7.

[33] Sterne JAC, Savović J, Page MJ, Elbers RG, Blencowe NS, Boutron I, et al. RoB 2: a revised tool for assessing risk of bias in randomised trials. BMJ. 2019;l4898. 10.1136/bmj.l4898. 10.1136/bmj.l4898 31462531

[34] Sterne JAC, Hernán MA, Reeves BC, Savović J, Berkman ND, Viswanathan M, et al. ROBINS-I: a tool for assessing risk of bias in non-randomised studies of interventions. BMJ. 2016;i4919. 10.1136/bmj.i4919. 10.1136/bmj.i4919 PMC5062054 27733354

[35] Guyatt G, Oxman AD, Akl EA, Kunz R, Vist G, Brozek J, et al. GRADE guidelines: 1. Introduction—GRADE evidence profiles and summary of findings tables. J Clin Epidemiol. 2011;64:383–94. 10.1016/j.jclinepi.2010.04.026. 21195583 10.1016/j.jclinepi.2010.04.026

[36] Dolveck F, Strazzulla A, Noel C, Aufaure S, Tarteret P, de Pontfarcy A, et al. COVID-19 among nursing home residents: results of an urgent pre-hospital intervention by a multidisciplinary task force. Brazilian J Infect Dis. 2021;25:101039. 10.1016/j.bjid.2020.11.004. 10.1016/j.bjid.2020.11.004 PMC7718835 33290728

[37] Li B, Yang Q. The effect of an ICU liaison nurse-led family‐centred transition intervention program in an adult ICU. Nurs Crit Care. 2023;28:435–45. 10.1111/nicc.12764. 35396917 10.1111/nicc.12764

[38] Mottaghi K, Hasanvand S, Goudarzi F, Heidarizadeh K, Ebrahimzadeh F. The role of the ICU liaison nurse services on anxiety in family caregivers of patients after ICU discharge during COVID-19 pandemic: a randomized controlled trial. BMC Nurs. 2022. 10.1186/s12912-022-01034-6. 36088385 10.1186/s12912-022-01034-6 PMC9464053

[39] Zhang X, Wang W, Zhao X, Cheng H, Song Y, Song X. Implementing caregiver management measures in general hospitals to prevent the COVID -19 pandemic. Nurs Open. 2023;10:2983–90. 10.1002/nop2.1542. 36528877 10.1002/nop2.1542 PMC9878028

[40] Calamassi D, Li Vigni ML, Fumagalli C, Gheri F, Pomponi GP, Bambi S. The listening to music tuned to 440 hz versus 432 hz to reduce anxiety and stress in emergency nurses during the COVID-19 pandemic: a double-blind, randomized controlled pilot study. Acta Biomed. 2022;93:e2022149. 10.23750/abm.v93is2.12915. 35545982 10.23750/abm.v93iS2.12915 PMC9534204

[41] Mohanty S, Lakkireddy D, Trivedi C, MacDonald B, Mayedo A, Della Rocca DG, et al. Creating a safe workplace by universal testing of SARS-CoV-2 infection in patients and healthcare workers in the electrophysiology unit having no symptoms of COVID-19: a multi-center experience. J Interventional Cardiac Electrophysiol. 2021;62:171–6. 10.1101/2020.07.14.20153494. 10.1007/s10840-020-00886-9 PMC7529320 33006086

[42] Ohta R, Ryu Y, Sano C. Effects of implementation of infection control measures against COVID-19 on the Condition of Japanese rural nursing homes. IJERPH. 2021;18:5805. 10.3390/ijerph18115805. 34071413 10.3390/ijerph18115805 PMC8198000

[43] Stemler J, Kramer T, Dimitriou V, Wieland U, Schumacher S, Sprute R, et al. Mobile PCR-based surveillance for SARS-CoV-2 to reduce visiting restrictions in nursing homes during the COVID-19 pandemic: a pilot study. Infection. 2022;50:607–16. 10.1007/s15010-021-01716-4. 34669164 10.1007/s15010-021-01716-4 PMC8527812

[44] Teixeira Mendes E, Neto DGPV, Ferreira GM, Valença IN, Lima MPJS, de Freitas MFMB, et al. Impact of COVID-19 RT-PCR testing of asymptomatic health care workers on absenteeism and hospital transmission during the pandemic. Am J Infect Control. 2023;51:248–54. 10.1016/j.ajic.2022.10.014. 36375707 10.1016/j.ajic.2022.10.014 PMC9671504

[45] Page MJ, McKenzie JE, Bossuyt PM, Boutron I, Hoffmann TC, Mulrow CD, et al. The PRISMA 2020 statement: an updated guideline for reporting systematic reviews. BMJ. 2021;n71. 10.1136/bmj.n71. 10.1136/bmj.n71 PMC8005924 33782057

[46] Goldsworthy S. The influence of simulation in predicting intent to stay, among critical care nurses. CJCCN. 2021;32:5–13. 10.5737/23688653-322513.

[47] Kumar N, Jin Y. Impact of nurses’ emotional labour on job stress and emotional exhaustion amid COVID-19: the role of instrumental support and coaching leadership as moderators. J Nurs Manage. 2022;30:2620–32. 10.1111/jonm.13818. 10.1111/jonm.13818 PMC9539243 36181253

[48] Nijland JWHM, Veling W, Lestestuiver BP, van Driel CMG. Virtual reality relaxation for reducing perceived stress of Intensive Care nurses during the COVID-19 pandemic. Front Psychol. 2021. 10.3389/fpsyg.2021.706527. 34659021 10.3389/fpsyg.2021.706527 PMC8511693

[49] Piscitello GM, Lamadrid VJ, Post Z, Kaur R, Gulczynski B, Baldeo R, et al. The Effect of Triggered Palliative Medicine consults on Nurse Moral Distress in the Medical Intensive Care Unit. Am J Hosp Palliat Care. 2022;39:1039–45. 10.1177/10499091211049398. 34587825 10.1177/10499091211049398

[50] Chang Y-C, Hsu M-C, Ouyang W-C. Effects of Integrated Workplace Violence Management Intervention on Occupational coping Self-Efficacy, goal commitment, attitudes, and confidence in Emergency Department nurses: a cluster-randomized controlled trial. IJERPH. 2022;19:2835. 10.3390/ijerph19052835. 35270527 10.3390/ijerph19052835 PMC8910583

[51] Goktas S, Gezginci E, Kartal H. The effects of motivational messages sent to emergency nurses during the COVID-19 pandemic on job satisfaction, Compassion fatigue, and communication skills: a Randomized Controlled Trial. J Emerg Nurs. 2022;48:547–58. 10.1016/j.jen.2022.06.001. 35864005 10.1016/j.jen.2022.06.001 PMC9226325

[52] Haugland WA, Crenshaw JT, Gilder RE. Implementing a Resilience Bundle for Emergency nurses: an evidence-based Practice Project. J Emerg Nurs. 2023;49:40–9. 10.1016/j.jen.2022.08.009. 36184334 10.1016/j.jen.2022.08.009 PMC9534550

[53] Hung C-L, Lin Y-L, Chou C-M, Wang C-J. Efficacy of aromatherapy at relieving the Work-Related Stress of Nursing Staff from various hospital departments during COVID-19. Healthcare. 2023;11:157. 10.3390/healthcare11020157. 36673525 10.3390/healthcare11020157 PMC9859127

[54] Rosamond RL, Giarratano G, Orlando S, Sumner J, Devier D, McDaniel LS, Wardell DW. Healing touch: a strategy for Acute Care nurses’ stress reduction. J Holist Nurs. 2023;41:347–59. 10.1177/08980101221142193. 36714962 10.1177/08980101221142193

[55] Zhang Y, Tang M, Zhou Y. Holistic sleep improvement strategies for frontline nurses served during a public health emergency (COVID -19) in Wuhan, China: a quasi‐experimental study. Nurs Open. 2023;10:1471–81. 10.1002/nop2.1397. 36209473 10.1002/nop2.1397 PMC9874611

[56] Pagador F, Barone M, Manoukian M, Xu W, Kim L. Effective holistic approaches to reducing nurse stress and burnout during COVID-19. AJN. Am J Nurs. 2022;122:40–7. 10.1097/01.NAJ.0000830744.96819.dc. 35447650 10.1097/01.NAJ.0000830744.96819.dc

[57] Ilmarinen J, Rantanen J. Promotion of work ability during ageing. Am J Ind Med. 1999;Suppl 1:21–3. 10.1002/(sici)1097-0274(199909)36. 10519773 10.1002/(sici)1097-0274(199909)36:1+<21::aid-ajim8>3.0.co;2-s

[58] Lippke S, Hessel A. Verhaltens- Und Verhältnisinterventionen in Der Prävention: Metaanalytische Befunde Und Implikationen. [Behavioural and relationship interventions in prevention: meta-analytical findings and implications]. PR. 2018;30:121–32. 10.5414/PRX0533.

[59] Aust B, Møller JL, Nordentoft M, Frydendall KB, Bengtsen E, Jensen AB, et al. How effective are organizational-level interventions in improving the psychosocial work environment, health, and retention of workers? A systematic overview of systematic reviews. Scand J Work Environ Health. 2023;49:315–29. 10.5271/sjweh.4097. 37158211 10.5271/sjweh.4097 PMC10713994

[60] Demerouti E, Bakker AB. Job demands-resources theory in times of crises: New propositions. Organizational Psychol Rev. 2023;13:209–36. 10.1177/20413866221135022.

[61] Parker SK, Jorritsma K. Good work design for all: multiple pathways to making a difference. Eur J Work Organizational Psychol. 2021;30:456–68. 10.1080/1359432X.2020.1860121.

[62] Eisenberger R, Rhoades Shanock L, Wen X. Perceived organizational support: why Caring about employees counts. Annu Rev Organ Psychol Organ Behav. 2020;7:101–24. 10.1146/annurev-orgpsych-012119-044917.

[63] Beehr TA, Jex SM, Stacy BA, Murray MA. Work stressors and coworker support as predictors of individual strain and job performance. J Organiz Behav. 2000;21:391–405. https://doi.org/10.1002/(SICI)1099-1379(200006)21:4<391::AID-JOB15>3.0.CO;2-9.

[64] Amason P, Allen MW, Holmes SA. Social support and acculturative stress in the multicultural workplace. J Appl Communication Res. 1999;27:310–34. 10.1080/00909889909365543.

[65] Daniels RA, Miller LA, Mian MZ, Black S. One size does NOT fit all: understanding differences in perceived organizational support during the COVID-19 pandemic. Bus Soc Rev. 2022;127:193–222. 10.1111/basr.12256.

[66] Zijlstra FRH. Effort as energy regulation. In: Battmann W, Dutke S, editors. Processes of the molar regulation of behavior. Berlin, Düsseldorf: Pabst Science Publ;: Lengerich; 1996. pp. 219–35.

[67] Meijman TF, Mulder G. Psychological aspects of workload. In: Drenth PJD, Thierry H, editors. A handbook of work and organizational psychology: volume 2: work psychology. 2nd ed. Hoboken: Psychology; 1998. pp. 5–33.

[68] Wendsche J, Ghadiri A, van Bengsch A, Wegge J. Antecedents and outcomes of nurses’ rest break organization: a scoping review. Int J Nurs Stud. 2017;75:65–80. 10.1016/j.ijnurstu.2017.07.005. 28750245 10.1016/j.ijnurstu.2017.07.005

[69] Cropanzano R, Mitchell MS. Social Exchange Theory: an Interdisciplinary Review. J Manag. 2005;31:874–900. 10.1177/0149206305279602.

[70] Schmucker R. Arbeitsbedingungen in Pflegeberufen. [Working conditions in nursing professions]. In: Jacobs K, Kuhlmey A, editors. Pflege-Report 2019; 2020. pp. 49–60. 10.1007/978-3-662-58935-9_3

[71] Federal Ministry of Labour and Social Affairs. editor. Act on the implementation of measures of occupational safety and health to encourage improvements in the safety and health protection of workers at work: Arbeitsschutzgesetz, ArbSchG.

[72] Dirani KM, Abadi M, Alizadeh A, Barhate B, Garza RC, Gunasekara N, et al. Leadership competencies and the essential role of human resource development in times of crisis: a response to Covid-19 pandemic. Hum Resource Dev Int. 2020;23:380–94. 10.1080/13678868.2020.1780078.

[73] Nekoie-Moghadam M, Kurland L, Moosazadeh M, Ingrassia PL, Della Corte F, Djalali A. Tools and checklists used for the evaluation of Hospital Disaster preparedness: a systematic review. Disaster med Public Health prep. 2016;10:781–8. 10.1017/dmp.2016.30. 27231031 10.1017/dmp.2016.30

[74] Richter P, Hacker W. Belastung und Beanspruchung: Stress, Ermüdung und Burnout im Arbeitsleben. [Stress and strain: stress, fatigue and burnout at work]. 1998.

[75] Andel SA, Tedone AM, Shen W, Arvan ML. Safety implications of different forms of understaffing among nurses during the COVID-19 pandemic. J Adv Nurs. 2022;78:121–30. 10.1111/jan.14952. 34240461 10.1111/jan.14952 PMC8450811

[76] Auffenberg J, Becka D, Evans M, Kokott N, Schleicher S, Braun E. „Ich pflege wieder, wenn …“: Potenzialanalyse zur Berufsrückkehr und Arbeitszeitaufstockung von Pflegefachkräften. [‘I'll nurse again if …’: Potential analysis for returning to work and increasing the working hours of nursing staff]. Bremen

[77] Stab N, Hacker W. Stationsorganisation Im Krankenhaus: Entwicklung Und Erprobung eines kriteriengeleiteten Bewertungs- Und Gestaltungsverfahrens: Forschung Projekt F 2253. BAUA-Bericht. [Ward organisation in hospitals: development and testing of a criteria-based evaluation and design procedure. Research Project F 2253]; 2016.

[78] Roodbari H, Nielsen K, Axtell C, Peters SE, Sorensen G. Testing middle range theories in realist evaluation: a case of a participatory organisational intervention. IJWHM. 2022;15:694–710. 10.1108/IJWHM-12-2021-0219. 10.3390/ijerph18168360 PMC8394353 34444110

[79] Nielsen K, Christensen M. Positive participatory organizational interventions: a Multilevel Approach for creating healthy workplaces. Front Psychol. 2021;12:696245. 10.3389/fpsyg.2021.696245. 34262513 10.3389/fpsyg.2021.696245 PMC8273334

[80] Palacios-Ceña D, Fernández-Peña R, Ortega-López A, Fernández-Feito A, Bautista-Villaécija O, Rodrigo-Pedrosa O, et al. Long-term care facilities and nursing homes during the First Wave of the COVID-19 pandemic: a scoping review of the perspectives of professionals, families and residents. IJERPH. 2021;18:10099. 10.3390/ijerph181910099. 34639401 10.3390/ijerph181910099 PMC8508277

[81] UN news. WHO chief declares end to COVID-19 as a global health emergency; 05,05.2023.

