# Supplementary material for: Managing the work stress of inpatient nurses during the COVID-19 pandemic: a systematic review of organizational interventions
Source: BMC Nurs. 2024 Sep 27;23:691. doi: 10.1186/s12912-024-02358-1 (PMC11437825; doi:10.1186/s12912-024-02358-1)
Supplement: Supplementary file 3 — Supplementary Material 3: Zink et al._Supplementary material_research data. [file 12912_2024_2358_MOESM3_ESM.pdf]

Supplementary material

Content

Supplement A 2
Table S1. Search string construction: Sources of search string components 2
Table S2. Final search strings 3
Supplement B 12
Figure S1. Checklist for title-abstract and full-text screening 12
Table S3. Operationalisation of PICOS for title-abstract and full-text screening 12
Supplement C 15
Table S4. Detailed intervention description (TIDieR) 15
Supplement D 29
Table S5. RoB-2 29
Table S6. ROBINS- I 30
Supplement E 31
Table S7. Rationale of included studies (pandemic associated work stressors and strains) 31
Supplement F 32
Table S8. Table of Outcomes: Rest break organization 32
Table S9. Table of Outcomes: Further studies 39

Supplement A

Table S1. *Search string construction: Sources of search string components*

PICOS	Search string from source (different databases)	Source and reasoning
Inpatient nurses	(DE "health personnel") OR (TX "health personnel") OR (TX healthcare AND TX workers) OR (TX "healthcare workers") OR (DE "nurses") OR (TX nurses) OR (TX nurse) OR (DE "physicians") OR (TX physicians) OR (DE "Medical Staff, Hospital") OR (TX "healthcare professionals") OR (TX "healthcare staff") OR (TX "medical staff") OR (TX "care workers") OR (TX "care personnel") OR (TX "nursing staff") OR (TX "nursing personnel") OR (TX caregivers) OR (TX carer)	Schubert et al., 2021<br>- Similar project in inpatient care<br>- used for nurse specific and closely related words
	("care worker*" or "careworker*" or "care giver*" or "caregiver*" or "care provider*" or "careprovider*" or "nurse" or "nurses").ab,ti.<br>AND<br>("personnel" or "staff" or "work force" or "workforce" or "workplace" or "organi?ation*" or "job").ab,ti.	Heijkants et al., 2022<br>- Detailed search string of similar project (stationary care including nursing homes)<br>- used for nurses in nursing home related words
COVID-19	(coronavirus OR "corona virus" OR coronavirinae OR coronaviridae OR betacoronavirus<br>OR covid19 OR "covid 19" OR nCoV OR "CoV 2" OR CoV2 OR sarscov2 OR 2019nCoV OR<br>"novel CoV" OR "wuhan virus") OR ((wuhan OR hubei OR huanan) AND ("severe acute respiratory" OR pneumonia) AND (outbreak)) OR "Coronavirus"[Mesh] OR Coronavirus Infections"[Mesh] OR "COVID-19" [Supplementary Concept] OR "severe acute respiratory syndrome coronavirus 2" [Supplementary Concept] OR "Betacoronavirus"[Mesh])	Schubert et al., 2021<br>- broad search string containing many different spellings of COVID-19 and related words<br>- preferred over other search strings as they included keywords for vaccination or diagnostics<br>- versions for CINAHL, PsycInfo and Pubmed available<br>- used without modification
Organizational interventions	("intervention" or "training" or "program" or "programme").ab,ti.	Heijkants et al., 2022<br>- general terms for intervention
	(interven* or program* or initiative* or approach* or project* or strateg* or reorganis* or reorganiz* or "re-organis*" or "re-organiz*" or redesign or "re-design" or restructuring or re-structuring or policy or policies or regulation* or guidance or	Nicolakakis et al., 2022<br>- general terms for intervention and (re-)organization<br>- other general organizational level terms were added;

	guideline or standard or solution or change)	specific work organization characteristics were not added as they are already included in "intervention" and would only add non-intervention search results
Study design	("control* stud*" or "clinical trial*" or "random* control* trial*" or "cluster random* trial*" or "case control stud*" or "pre test posttest control group design" or "pretest" or "posttest" or "static group comparison" or "pilot stud*" or "quasiexperiment*" or "quasi experiment*" or "evaluat*" or "time series" or "time point*" or "repeated measur*").tw.	Heijkants et al., 2022<br>- used for experimental and quasiexperimental designs<br>- removed "evaluat*" as results exploded by this keyword (word used in almost every abstract)
	"randomized controlled trial"[pt] OR "controlled clinical trial"[pt] OR randomized[tiab] OR placebo[tiab] OR "drug therapy"[sh] OR randomly[tiab] OR trial[tiab] OR groups[tiab]	Cochrane filter for RCT (Lefebvre et al., 2022)
	Added MeSh terms and other standardized vocabulary; e.g., Controlled Before-After Studies"[MeSH] OR "Interrupted Time Series Analysis" [Mesh] OR "multiple time series"[tiab] OR "pre test*"[tiab] OR "post test*"[tiab])	

Table S2. *Final search strings*

PubMed	
**#1 Nurses**	("Nurses"[MeSH Major Topic] OR "Nursing Staff"[MeSH Major Topic] OR "Licensed Practical Nurses"[MeSH Major Topic] OR nurs*[Title] OR certified-nursing-assistant*[Title] OR care-worker*[Title] OR careworker*[Title] OR care-giver*[Title] OR "caregiver*"[Title] OR care-provider*[Title] OR "careprovider*"[Title] OR nursing-home-personnel[Title])
**#2 COVID-19**	((coronavirus OR "corona virus" OR coronavirinae OR coronaviridae OR betacoronavirus OR covid19 OR "covid 19" OR nCoV OR "CoV 2" OR CoV2 OR sarscov2 OR 2019nCoV OR "novel CoV" OR "wuhan virus") OR ((wuhan OR hubei OR huanan) AND ("severe acute respiratory" OR pneumonia) AND (outbreak)) OR "Coronavirus"[Mesh] OR "Coronavirus Infections"[Mesh] OR "COVID-19" [Supplementary Concept] OR "severe acute respiratory syndrome coronavirus 2" [Supplementary Concept] OR "Betacoronavirus"[Mesh])

**#3 organizational intervention**<br>Keywords for<br>- Intervention<br>- Job design<br>- (Re-)Organisation<br>- Crisis preparedness as organizational task in times of crisis	("intervention"[tiab] OR "training"[tiab] OR "program"[tiab] OR "programme"[tiab] OR "approach"[tiab] OR "initative"[tiab] OR "project"[tiab] OR "adapt*"[tiab] OR "implement*"[tiab] OR work-redesign [tiab] OR job-redesign [tiab] OR work-design [tiab] OR job-design [tiab] OR "management"[tiab] OR "reorganis*"[tiab] OR "reorganiz*"[tiab] OR "re-organis*"[tiab] OR "re-organiz*"[tiab] OR "restructur*"[tiab] OR "re-structur*"[tiab] OR ("crisis"[tiab] AND ("preparedness"[tiab] OR "readiness"[tiab] OR "prevention")) OR "Organization and Administration" [MeSh])
**#4 Study design**<br>Interventionsstudien<br>Experiments (RCT,…)<br>Quasi-experiments with at least one Pre- and one Post-Measurement	("Controlled Before-After Studies"[MeSh] OR "Interrupted-Time-Series Analysis"[MeSh] OR "Cross-Over Studies" [MeSh] OR randomized-controlled-trial[pt] OR controlled-clinical-trial[pt] OR randomized[tiab] OR randomised[tiab]OR placebo[tiab] OR randomly[tiab] OR trial[tiab] OR groups[tiab] OR intervention-stud*[tiab] OR controlled-stud*[tiab] OR clinical-trial*[tiab] OR program-evaluat*[tiab] OR process-evaluat* [tiab] OR randomized-controlled-trial*[tiab] OR randomised-controlled-trial*[tiab] OR cluster-randomized-trial*[tiab] OR cluster-randomised-trial*[tiab] OR "pretest-posttest control-group"[tiab] OR "pretest*"[tiab] OR "posttest*"[tiab] OR static-group-comparison[tiab] OR "quasiexperiment*"[tiab] OR quasi-experiment*[tiab] OR time-series[tiab] OR time-point*[tiab] OR repeated-measur*[tiab] OR before-and-after [tiab] OR pre-intervention [tiab] OR post-intervention [tiab] OR multiple-time-series[tiab] OR "pre-test*"[tiab] OR "post-test*"[tiab])
**#5 Filter: timeframe**<br>2020 - 2023	(2020/01/01:2023/12/31[Date - Publication])
**#6 Filter: Sprache**<br>Deutsch und Englisch	("english"[Language] OR "german"[Language])
**#7 Combination**	#1 AND #2 AND #3 AND 4# AND #5 AND #6

CINAHL (EBSCO)	
**#1 nurses**	(MM "Nurses+") OR (MM "Nursing Staff, Hospital") OR (MM "Nursing Home Personnel") OR (MM "Nursing Assistants") OR (TI "nurs*") OR (TI "care worker*") OR (TI "careworker*") OR (TI "care giver*") OR (TI "caregiver*") OR (TI "careprovider*") OR (TI "care provider*") OR (TI "certified nursing assistant*") OR (TI "licensed practical nurs*")
**#2 COVID-19**	(TX coronavir*) OR (TX corona virus*) OR (TX betacoronavir*) OR (TX "covid 19") OR (TX nCoV) OR (TX "CoV 2") OR (TX CoV2) OR (TX sarscov2) OR (TX 2019nCoV) OR (TX "2019 novel coronavirus*") OR (TX "2019 novel CoV") OR (TX "wuhan virus*") OR (TX (wuhan OR hubei OR huanan) AND (severe acute respiratory OR pneumonia*) AND (outbreak*)) OR (DE "Coronavirus")

**#3 organizational intervention**<br>Keywords for<br>- Intervention<br>- Job design<br>- (Re-)Organisation<br>- Crisis preparedness as organizational task in times of crisis	(MM "Work Redesign") OR (MM "Management+") OR (TI "intervention") OR (TI "training") OR (TI "program") OR (TI "programme") OR (TI "approach") OR (TI "implement*") OR (TI "initative") OR (TI "project") OR (TI "adapt*") OR (TI "work redesign") OR (TI "work design") OR (TI "job design") OR (TI "job redesign") OR (TI "management") OR (TI "reorganis*") OR (TI "reorganiz*") OR (TI "re-organis*") OR (TI "re-organiz*") OR (TI "restructur*") OR (TI "re-structur*") OR (TI "workplace" AND (TI "organis*" OR TI "organiz*")) OR (TI "crisis" AND (TI "preparedness" OR TI "readiness" OR TI "prevention"))<br>OR (AB "intervention") OR (AB "training") OR (AB "program") OR (AB "programme") OR (AB "approach") OR (AB "initative") OR (AB "project") OR (AB "adapt*") OR (AB "implement*") OR (AB "work redesign") OR (AB "work design") OR (AB "job design") OR (AB "job redesign") OR (AB "management") OR (AB "reorganis*") OR (AB "reorganiz*") OR (AB "re-organis*") OR (AB "re-organiz*") OR (AB "restructur*") OR (AB "re-structur*") OR (AB "workplace" AND (AB "organis*" OR AB "organiz*")) OR (AB "crisis" AND (AB "preparedness" OR AB "readiness" OR AB "prevention"))
**#4 Study design**<br>Intervention studies<br>- Experiments (RCT,…)<br>- Quasi-experiments with at least one Pre- and one Post-measurement	(PT "randomized controlled trial") OR (PT controlled-clinical-trial) OR (MH "Experimental Studies+") OR (MH "Quasi-Experimental Studies+") OR (MH "Controlled Before-After Studies") OR (MH "Interrupted Time Series Analysis") OR (MH "Clinical Trials") OR (MH "Crossover Design") OR (MH "Pretest-Posttest Design+") OR<br><br>(TI "randomi#ed") OR (TI "placebo") OR (TI "randomly") OR (TI "trial") OR (TI "groups") OR (TI "intervention stud*") OR (TI "controlled stud*") OR (TI "clinical trial*") OR (TI "program evaluat*") OR (TI ("process*" N0 "evaluat*")) OR (TI "randomi#ed controlled trial*") OR (TI "cluster randomi#ed trial*") OR (TI "pretest posttest control group") OR (TI "pretest*") OR (TI "posttest*") OR (TI "static group comparison") OR (TI "quasiexperiment*") OR (TI "quasi experiment*") OR (TI "time-series") OR (TI "time point*") OR (TI "repeated measur*") OR (TI "multiple time series") OR (TI "pre-test*") OR (TI "post-test*") OR (TI "before and after") OR (TI "pre intervention") OR (TI "post intervention") OR<br><br>(AB "randomi#ed") OR (AB "placebo") OR (AB "randomly") OR (AB "trial") OR (AB "groups") OR (AB "intervention stud*") OR (AB "controlled stud*") OR (AB "clinical trial*") OR (AB "program evaluat*") OR (AB ("process*" N0 "evaluat*")) OR (AB "randomi#ed controlled trial*") OR (AB "cluster randomi#ed trial*") OR (AB "pretest posttest control group")

	OR (AB "pretest*") OR (AB "posttest*") OR (AB "static group comparison") OR (AB "quasiexperiment*") OR (AB "quasi-experiment*") OR (AB "time-series") OR (AB "time point*") OR (AB "repeated measur*") OR (AB "multiple time series") OR (AB "pre-test*") OR (AB "post-test*") OR (AB "before and after") OR (AB "pre intervention") OR (AB "post intervention")
**#5 Combination**	#1 AND #2 AND #3 AND 4#
Filter time frame	2020-2023
Filter language	Deutsch und Englisch

PsycINFO/ARTICLES (EBSCO)	
**#1 nurses**	((MM "Nurses") OR (TI "nursing staff ") OR (TI "Nursing Home Personnel" OR (MM "Nursing Homes" AND MM "Personnel")) OR (TI "nursing assistant*") OR (TI "nurs*") OR (TI "care worker*") OR (TI "careworker*") OR (TI "care giver*") OR (TI "caregiver*") OR (TI "careprovider*") OR (TI "care provider*") OR (TI "certified nursing assistant*") OR (TI "licensed practical nurs*"))
**#2 COVID-19**	((TX coronavir*) OR (TX corona virus*) OR (TX betacoronavir*) OR (TX "covid 19") OR (TX nCoV) OR (TX "CoV 2") OR (TX CoV2) OR (TX sarscov2) OR (TX 2019nCoV) OR (TX "2019 novel coronavirus*") OR (TX "2019 novel CoV") OR (TX "wuhan virus*") OR (TX (wuhan OR hubei OR huanan) AND (severe acute respiratory OR pneumonia*) AND (outbreak*)) OR (DE "Coronavirus"))
**#3 organizational intervention**<br>Keywords for<br>- Intervention<br>- Job design<br>- (Re-)Organisation<br>- Crisis preparedness as organizational task in times of crisis	((MM "Intervention" OR MM "Crisis Intervention" OR MM "Group Intervention" OR MM "Workplace Intervention") OR (MM "Personnel Training") OR (MM "Human Resource Management" OR MM "Career Development" OR MM "Employee Benefits" OR MM "Job Analysis" OR MM "Labor Management Relations" OR MM "Outsourcing" OR MM "Personnel Evaluation" OR MM "Personnel Recruitment" OR MM "Personnel Selection" OR MM "Personnel Termination") OR (MM "Management" OR MM "Management Planning" OR MM "Risk Management" OR MM "Self-Management" OR MM "Stress Management" OR MM "Time Management") OR<br>(TI "intervention") OR (TI "training") OR (TI "program") OR (TI "programme") OR (TI "approach") OR (TI "implement*") OR (TI "work redesign") OR (TI "job redesign") OR (TI "work design") OR (TI "job design") OR (TI "management") OR (TI "reorganis*") OR (TI "reorganiz*") OR (TI "re-organis*") OR (TI "re-organiz*") OR (TI "restructur*") OR (TI "re-structur*") OR (TI "initative") OR (TI "project") OR (TI "adapt*") OR (TI "crisis" AND (TI "preparedness" OR TI "readiness" OR TI "prevention")) OR (TI "workplace" AND (TI "organis*" OR TI "organiz*")) OR<br>(AB "intervention") OR (AB "training") OR (AB "program") OR (AB "programme") OR (AB "approach") OR (AB "implement*") OR (AB "work redesign") OR (AB "job

	redesign") OR (AB "work design") OR (AB "job design") OR (AB "management") OR (AB "reorganis*") OR (AB "reorganiz*") OR (AB "re-organis*") OR (AB "re-organiz*") OR (AB "restructur*") OR (AB "re-structur*") OR (AB "initative") OR (AB "project") OR (AB "adapt*") OR (AB "crisis" AND (AB "preparedness" OR AB "readiness" OR AB "prevention")) OR (AB "workplace" AND (AB "organis*" OR AB "organiz*")))
**#4 Study design**<br>Interventionsstudien<br>- Experiments (RCT,…)<br>Quasi-experiments mit mind. einer Pre- und einer Post-Messung	((DE "Experimental Design") OR (DE "Randomized Controlled Trials") OR (DE "Evidence Based Practice") OR (DE "Treatment Effectiveness Evaluation") OR (DE "Between Groups Design") OR (DE "Followup Studies") OR (DE "Longitudinal Studies") OR (DE "Repeated Measures" OR (DE "Single-Case Experimental Design") OR (DE "Program Evaluation") OR<br>(TI "randomi#ed") OR (TI "placebo") OR (TI "randomly") OR (TI "trial") OR (TI "groups") OR (TI "intervention stud*") OR (TI "controlled stud*") OR (TI "clinical trial*") OR (TI "program evaluat*") OR (TI ("process*"N0"evaluat*")) OR (TI "randomi#ed controlled trial*") OR (TI "cluster randomi#ed trial*") OR (TI "pretest posttest control group") OR (TI "pretest*") OR (TI "posttest*") OR (TI "static group comparison") OR (TI "quasiexperiment*") OR (TI "quasi-experiment*") OR (TI "time-series") OR (TI "time point*") OR (TI "repeated measur*") OR (TI "controlled before-after") OR (TI "multiple time series") OR (TI "pre-test*") OR (TI "post-test*") OR (TI "before and after") OR (TI "pre intervention") OR (TI "post intervention") OR<br>(AB "randomi#ed") OR (AB "placebo") OR (AB "randomly") OR (AB "trial") OR (AB "groups") OR (AB "intervention stud*") OR (AB "controlled stud*") OR (AB "clinical trial*") OR (AB "program evaluat*") OR (TI ("process*"N0"evaluat*")) OR (AB "randomi#ed controlled trial*") OR (AB "cluster randomi#ed trial*") OR (AB "pretest posttest control group") OR (AB "pretest*") OR (AB "posttest*") OR (AB "static group comparison") OR (AB "quasiexperiment*") OR (AB "quasi experiment*") OR (AB "time-series") OR (AB "time point*") OR (AB "repeated measur*") OR (AB "controlled before-after") OR (AB "multiple time series") OR (AB "pre-test*") OR (AB "post-test*") OR (AB "before and after") OR (AB "pre intervention") OR (AB "post intervention"))
**#5 Combination**	#1 AND #2 AND #3 AND #4
Filter time frame	01/2020-2023
Filter language	Deutsch und Englisch

PsyArxiv	
**#1 Nurses**	("nurses" OR "nursing staff" OR "nursing home personnel" OR "nursing assistant*" OR "nurs*" OR "care worker*" OR "careworker*" OR "care giver*" OR "caregiver*" OR "careprovider*" OR "care provider*" OR "certified nursing assistant*" OR "licensed practical nurs*")
**#2 COVID-19**	("coronavir*" OR corona virus* OR betacoronavir* OR "covid 19" OR "nCoV" OR "CoV 2" OR "CoV2" OR "sarscov2" OR "2019nCoV" OR "2019 novel coronavirus*" OR "2019 novel CoV" OR "wuhan virus*" OR (("wuhan" OR "hubei" OR "Huanan") AND ("severe acute respiratory" OR "pneumonia*") AND ("outbreak*")) OR "Coronavirus")
**#3 organisationale Intervention (Allgemein)**	("intervention" OR "training" OR "program" OR "programme" OR "approach" OR "implement*" OR "initative" OR "project" OR "adapt*" OR "work redesign" OR "work design" OR "job redesign" OR "job design" OR "management" OR "reorganis*" OR "reorganiz*" OR "re organis*" OR "re organiz*" OR "restructur*" OR "re structur*" OR ("crisis" AND ("preparedness" OR "readiness" OR "prevention")) OR ("workplace" AND ("organiz*" OR "organis*")))
**#4 Study design**	(("experimental stud*" OR "quasiexperiment*" OR "quasi experiment*" OR "randomized controlled trial" OR "controlled clinical trial" OR "randomi?ed" OR "placebo" OR "randomly" OR "trial" OR "groups" OR "interventions stud*" OR "controlled stud*" OR "clinical trial*" OR "program* evaluat*" OR "process evaluat*" OR "randomi?ed controlled trial" OR "cluster randomi?ed trial" OR "pretest posttest control group" OR "pretest*" OR "posttest" OR "pre test" OR "post test" OR "static group comparison" OR "time series" OR "time point" OR "repeated measur*" OR "multiple time series" OR "before and after" OR "pre intervention" OR "post intervention" OR "before after" OR "interrupted time series" OR "clinical trial*"))
**#5 Combination**	#1 AND #2 AND #3 AND #4
**Filter Zeitraum**	Not implemented.
**Filter Sprache**	Not implemented due to lacking instructions..

medRxiv Script in RStudio
#Problem: When searching for the combined search strings nurses, COVID, orgaInt, or StudyDesign we get an error output when the searchstrings themselves contain lists<br>#First possibility: Simplify search strings --> no lists (=AND) in nurses, COVID, orgaInt, or StudyDesign<br>#before running the skript make sure to adapt setwd and to change to_date in the searches to today!<br><br>setwd("~/RStudio Exports")<br>library(medrxivr)<br>preprint_data <- mx_snapshot()<br>auto_caps = TRUE<br><br>nurses <- c ("nurs*", "nursing staff", "nursing home personnel", "nursing assistant", "care worker*", "careworker*", "care giver*", "caregiver*", "careprovider*", "care provider*", "certified nursing assistant*", "licensed practical nurse*")

```
	COVID <- c("coronavir*", "corona virus", "betacoronavir*", "covid 19", "nCoV", "CoV 2", "sarscov2", "2019nCoV", "2019 novel coronavirus", "2019 novel CoV", "wuhan virus", "Coronavirus")

	orgaInt <- c("intervention", "training", "program", "programme","approach", "implement", "initiative", "project", "adapt", "work redesign",  "job redesign", "work design", "job design", "management", "reorgani*","re-organi*", "restructur", "re-structur", "crisis preparedness", "crisis readiness", "crisis prevention", "workplace organiz*", "workplace organis*")

	StudyDesign <- c("experimental stud", "quasi experiment*", "quasi-experiment*", "randomi*ed controlled trial", "controlled clinical trial", "randomi*", "placebo", "randomly", "trial", "groups", "interventions stud", "controlled stud", "clinical trial", "program* evaluat", "process evaluat*", "cluster randomi*ed trial", "pretest posttest control group", "pretest", "posttest", "pre test", "post test", "static group comparison", "time series", "time point", "repeated measur*","multiple time series", "before and after", "pre intervention", "post intervention", "before-after", "interrupted time series", "clinical trial")

	myquery <- list(nurses, COVID, orgaInt, StudyDesign)
	mx_results <- mx_search(data = mx_snapshot(),
				query = myquery,
				fields = c("title","abstract"),
				from_date = "2020-01-01",
				to_date = "2023-03-15"
				)
	mx_export(data = mx_results,
		file = "mx_search_results.bib")

	#Favored Alternative: Search by combining 4 Search Results: nurses AND COVID-19 AND organizational interventions AND study designs
	setwd("~/RStudio Exports")
	library(medrxivr)
	preprint_data <- mx_snapshot()

	auto_caps = TRUE

	 nurses <- c ("nurs*", "nursing staff", "nursing home personnel", "nursing assistant", "care worker*", "careworker*", "care giver*", "caregiver*", "careprovider*", "care provider*", "certified nursing assistant*", "licensed practical nurse*")
	 nursesquery <- list(nurses)
	 mx_resultsNURSES <- mx_search(data = mx_snapshot(),
				query = nursesquery,
				fields = c("title","abstract"),
				from_date = "2020-01-01",
				to_date = "2023-03-15"
				)

	 #OutbreakTerm = ("severe acute respiratory", "pneumonia", "outbreak") AND ("wuhan", "hubei","Huanan")
	 #search for CovidNames OR OutbreakTerm --> needs seperate search OutbreakTerm --> combine seperate search outputs for OutbreakTerm and CovidNames
	 outbreak <- c("severe acute respiratory", "pneumonia", "outbreak")
	 OutbreakORIGIN <- c("wuhan", "hubei","Huanan")
	 OutbreakTermquery <- list (outbreak, OutbreakORIGIN)
```

```
        mx_resultsOUTBREAK <- mx_search(data = mx_snapshot(),
                              query = OutbreakTermquery,
                              fields = c("title","abstract"),
                              from_date = "2020-01-01",
                              to_date = "2023-03-15"
                              )
       CovidNames <-c("coronavir*", "corona virus", "betacoronavir*", "covid 19",
"nCoV", "CoV 2", "sarscov2", "2019nCoV", "2019 novel coronavirus", "2019 novel CoV",
"wuhan virus", "Coronavirus")
        mx_resultsCOVID19 <- mx_search(data = mx_snapshot(),
                              query = CovidNames,
                              fields = c("title","abstract"),
                              from_date = "2020-01-01",
                              to_date = "2023-03-15"
                              )
        #union both searches without duplicates:
        library(dplyr)
        COVID <-union(mx_resultsOUTBREAK, mx_resultsCOVID19)

       #for oganizational Interventions combine three searches (=with OR), namely
Crisis Preparedness, WorkplaceOrganization, and orgaInt
        Crisis <-c("crisis")
        Preparedness <-c("preparedness", "readiness", "prevention")
        CrisisPreparednessQuery <-list(Crisis, Preparedness)
         mx_resultsCRISIS <- mx_search(data = mx_snapshot(),
                                   query = CrisisPreparednessQuery,
                                   fields = c("title","abstract"),
                                   from_date = "2020-01-01",
                                   to_date = "2023-03-15"
                                   )
        Workplace <-c("workplace")
        Organization <-c("organiz*", "organis*")
        WorkplaceOrganizationQuery <-list(Workplace, Organization)
         mx_resultsWORKPLACE <- mx_search(data = mx_snapshot(),
                                  query = WorkplaceOrganizationQuery,
                                  fields = c("title","abstract"),
                                  from_date = "2020-01-01",
                                  to_date = "2023-03-15"
                                  )
        orgaInt <- c("intervention", "training", "program", "programme","approach",
"implement", "initiative", "project", "adapt", "work redesign",  "job redesign", "work design",
"job design", "management", "reorgani*","re-organi*", "restructur", "re-structur")
         mx_resultsINTERVENTION <- mx_search(data = mx_snapshot(),
                                  query = orgaInt,
                                  fields = c("title","abstract"),
                                  from_date = "2020-01-01",
                                  to_date = "2023-03-15"
                                  )
        #combine all searches (with OR!) by using union (combines without duplicates)
        Organization <-union(mx_resultsCRISIS,mx_resultsWORKPLACE)
        OrganizationalInterventions <-union(Organization,mx_resultsINTERVENTION)

       #for Study Designs only one search for keywords
        StudyDesign <- c("experimental stud", "quasi experiment*", "quasi-experiment*",
"randomi*ed controlled trial", "controlled clinical trial", "randomi*", "placebo", "randomly",
```

```
"trial", "groups", "interventions stud", "controlled stud", "clinical trial", "program* evaluat",
"process evaluat*", "cluster randomi*ed trial", "pretest posttest control group", "pretest",
"posttest", "pre test", "post test", "static group comparison", "time series", "time point",
"repeated measur","multiple time series", "before and after", "pre intervention", "post
intervention", "before-after", "interrupted time series", "clinical trial")
            mx_resultsStudyDesign <- mx_search(data = mx_snapshot(),
                                        query = StudyDesign,
                                        fields = c("title","abstract"),
                                        from_date = "2020-01-01",
                                        to_date = "2023-03-15"
                                        )
        #Create an Intersection (= with AND!) of the Tables
mx_resultsNURSES,COVID,OrganizationalInterventions, and mx_resultsStudyDesign
          Intersect1 <- intersect(mx_resultsNURSES,COVID)
          Intersect2 <- intersect(OrganizationalInterventions,mx_resultsStudyDesign)
          QueryRESULT <-intersect(Intersect1,Intersect2)

        #export as bib
          mx_export(data = QueryRESULT,
                file = "mx_search_results.bib")
```

Supplement B

Figure S1*. Checklist for title-abstract and full-text screening*

Can you answer the question with "Yes"? > No > Reason for exclusion > Coding

**Question & Examples**	**Reason for exclusion**	**Coding**
Is the article English or German?	Not English or German language	1
Is the article a primary study, i.e. collected data or produced new information?	No primary study	2
Does the study population explicitly include nurses who work in inpatient care, i.e. licensed practical nurses working in COVID-19 department?	Wrong population	3
Does the study explicitly mention COVID-19 related challenges as reason for the intervention, i.e. work overload due to sickness absence and increased hospitalization rates?	No pandemic-related challenge addressed	4
Does the study proactively implement an intervention?	No Intervention	5
Does the implemented intervention change how inpatient nurses' work is designed, managed, or organized, i.e. by redesigning shift schedules?	No organizational level intervention	6
Was the study an experiment (RCT), or a quasi-experiment with at least one pre and post measurement? i.e. Crossover trial, before and after study design, …	Wrong study design	7
Does the study report final results?	Pending study results	8

Note: Not peer-reviewed is not a reason for exclusion but has to be noted (Label "non-peer-reviewed") since preregistration studies are not commonly peer-reviewed.

In case you encounter Duplicates please "Exclude" the entry with abbreviated Author names or with less information that the other entry. Then add Label: "Duplicate!"

Table S3*. Operationalisation of PICOS for title-abstract and full-text screening*

PIC(O)S	Definition	Further explanations
Nurses working in inpatient care	1. *Nurses*: Provide direct care for patients or clients in need for care due to the effects of ageing, illness, disease, injuries or other physical or mental impairments (WHO, 2019).<br><br>Nursing professionals (e.g., professional nurse, specialist nurse)<br>Qualification: Received formal training at a higher educational institution of nursing<br>Work activities include: Planning and management of the care of patients (incl. supervision of other health care workers), practical application of preventive and curative interventions in clinical and community	Include studies with nursing students (either together with professional nurses or as single target population) only if they shared the same work context as professional, namely an inpatient care facility where the intervention was implemented.

	settings, either working autonomously or in teams with medical doctors and others<br><br>Nursing associate professionals (e.g., assistant nurses, specialist nurses)<br>Qualification: Acquired relevant skills and knowledge through either study or extensive on-the-job training<br>Work activities include: basic nursing and personal care, monitoring of patients' conditions, implementation of care, treatment or referral plans, providing health advice for patients and patients' families<br><br>Healthcare assistants<br>Qualification: extensive healthcare knowledge or training not required<br>Work activities: routine care, support and assistance to patients or residents<br><br>2. *Inpatient care*: Nurses have to work in a facility where care is delivered to inpatients, e.g., hospitals or nursing homes. An inpatient is "[a] person who is formally admitted to a health-care facility and who is discharged after one or more days" (WHO, 2009, p. 240).	Include studies with healthcare workers in general, if they distinguished between the different occupation groups (physicians, nurses, pharmacists, etc.) in their results.
Organizational Intervention	Interventions have to be proactively planned and goal oriented while explicitly addressing pandemic-associated challenges of inpatient nurses.<br><br>Pandemic-associated challenges are either pre-pandemic work stressors (e.g., work overload) or work stressors that arose throughout the pandemic (e.g., lack of PPE).<br><br>Organizational level interventions shape the way nurses' work is organized, designed and managed from a "top-down" perspective (Parker & Jorritsma, 2021). Such direct top down modifications to work aspects outside of the individual person include the (re-)design of work tasks and activities (e.g., responsibilities, task quantity), of resources (e.g., tools, technologies, human resources), of time structures (e.g., shift schedules, rosters), and of the physical work environment (e.g., unit organization)(Holden et al., 2013).	Include infection control interventions if they are initiated by the health institution (hospital or nursing home) in addition to public health regulations, e.g., preventative unit wide testing.<br><br>Include interventions such as the organization of health circles or task forces to improve work design by direct participation of nurses (= new responsibilities or activities for nurses)<br><br>Exclude infection control interventions if they are implemented on a larger cross-institutional scale as responsibility of public health.

		Exclude interventions if they aim to influence work design indirectly through awareness raising or trainings on work design in order to inspire bottom-up informal actions and emergent social processes (Parker & Jorritsma, 2021).
Comparison	compared to no or to other intervention	
Interventional study design	interventional studies:<br>randomized controlled trials (RCT)<br>quasiexperimental studies with at least one pre- and one post-test (e.g., controlled before and after studies with or without control group, interrupted time series, etc.)	
Time Frame and Publication type	published between 01/2020 and 03/2022<br>published:<br>in journal (peer-reviewed)<br>preregistration database	Note: Do not exclude Grey Literature if it fits all Inclusion criteria. Label: "Grey Literature"
Language	German or English	

Supplement C

Table S4. *Detailed intervention description (TIDieR)*

Inter-vention	Setting/ context	Work stressor/ rationale	Prevention approach	Modified work system components	Duration, materials, procedures, mode of delivery	Tailoring	Modifica-tions	Fidelity
**Integrated Workplace Violence Management** (Chang et al., 2022)	emergency nurses in emergency department in designated COVID-19 hospital	nurses who provide care for patients with COVID-19 experience higher rates of physical violence and verbal abuse than who do not<br><br>→ a**im/ hypothesis:** "Workplace violence management intervention" in conjunction with routine in-service class lead to improvements in outcomes measured on ED nurses	secondary	time<br>physical environment<br>**X resources**<br>**X activities**<br>social	**Multi-level intervention**<br>**A) Person-oriented part: Workplace violence (WPV) class**<br>- in addition to standard 1-hour in-service class about hospital safety policies and procedures<br>- held by a psychiatrist and a social worker, using "proactive questioning, role-plays, scenario examples based on actual WPV, in various communication exercises, discussions, and debriefing." (p.5)<br>- 12 sessions (> 1hr) with 12 components through interactive video conferences: *Awareness of WPV* (identification of high-risk patients/visitors, motivations of perpetrators, causal factors for PVV, possible triggers of violence, cues to impending aggression), *What to do* (interaction, management, prevention, and post-incident action, such as danger assessment, communication skills for a potentially threatening situation, problem solving, conflict management, anger management), *Competences* (assertiveness techniques, engaging in more complicated interactions, and proactive violence management)	no	no	no

Inter-vention	Setting/ context	Work stressor/ rationale	Prevention approach	Modified work system components	Duration, materials, procedures, mode of delivery	Tailoring	Modifica-tions	Fidelity
					**B) job-oriented part of intervention**:<br>- regular team-briefing and feedback<br>- checklist for nurses with key components of the intervention			
**professional development simulation intervention** (Goldsworthy, 2021)	critical care nurses from different hospitals	critical shortages specifically of critical care nurses due to high hospitalization rates<br><br>→ **aim/ hypothesis:**<br>a) professional development intervention improves nurses' intent to stay, and<br>b) perceived organizational support mediates this relationship	secondary	time<br>physical environment<br>resources<br>**X activities**<br>social	**Multi-level intervention**<br><br>324-hr self-paced, critical care certificate program over a one-year period consisting; three parts:<br><br>**A) person-oriented part**<br>**1. Instructor-facilitated asynchronous online learning modules** (6 sessions, 315 hr)<br>- offered in an asynchronous format<br>- modules: introduction to e-learning, advanced pathophysiology, cardiac, respiratory, GI/ renal, neuro<br><br>**2. High-fidelity simulation course** (39 hr over two weekends)<br>- onsite instructor-facilitated by eight instructors with >25 years of experience<br>- prior to simulation instructors mentored the simulation team to run the case<br>- instructors evaluated and tested participants via competency-based checklist<br>- three stations to prepare participants (arrhythmia/12 lead ECG interpretation, management of mechanical ventilation and hemodynamic monitoring management)	no	no	no

Inter-vention	Setting/ context	Work stressor/ rationale	Prevention approach	Modified work system components	Duration, materials, procedures, mode of delivery	Tailoring	Modifica-tions	Fidelity
					- followed by nine critical cases (each case: pre-brief of 10 min, case duration of 20 min, post-test questions of 30 min): septic shock, respiratory distress/acute respiratory failure, hypovolemic shock, end of life (mock family conference), myocardial infarction, abdominal aortic aneurysm repair (AAA), hemodynamic instability, acute renal failure and head injury/trauma<br>- material for simulations: for each case pre-determined template (including learning objectives), pre-test questions, initial patient phase + evolving patient phase + conclusion phase; competency based checklists for evaluating and testing the students (created by a team of critical care experts)<br><br>**B) job-oriented approach**<br>**1. Preceptored practicum** (120 hr)<br>ten 12-hour shifts with implementation of new skills in adult critical care			
**Aromatherapy on ward** (Hung et al., 2023)	during COVID-19 surge<br><br>nursing staff from different units (palliative, general, ObGyn, ICU)	severe anxiety, stress, and post traumatic stress disorder symptoms of nursing staff during the COVID-19 pandemic	secondary	time<br>**X physical environment**<br>resources<br>activities<br>social	**Job-oriented approach**<br>Aroma oil diffusers were placed in different units where most work was done at backside of nursing counter to prevent undesirable effects on patients.<br>intervention carried out for 4 weeks twice every weekday (Friday-Monday) at 8:00-12:00 a.m. and 16:00-20:00 p.m.; dose: 5 drops of 100% bergamot peel essential oil (<46% limonene, <35% linalyl acetate, linalool <23%)	no	no	improved ventilation during COVID-19 may have hindered the intervention

Inter-vention	Setting/ context	Work stressor/ rationale	Prevention approach	Modified work system components	Duration, materials, procedures, mode of delivery	Tailoring	Modifica-tions	Fidelity
		→ aim/ **hypothesis:** aromatherapy to relieve the stress of nursing staff working during COVID-19			applied by ultrasonic diffuser for aroma evaporation			
**instrumental support and coaching leadership** (Kumar & Jin, 2022)	ICU nurses working with COVID-19 patients in 107 hospitals	increased job demands of nurses during the COVID-19 pandemic, excessive demands require inpatient nurses to perform emotional labor<br><br>→ aim/ **hypothesis:** a) instrumental support lessens the impact of surface acting on job stress in emergency (foster nurses' capabilities to tackle increased job demands) and	Primary and secondary	time<br>physical environment<br>**X resources**<br>**X activities**<br>**X social**	**Job-oriented approach**<br>two levels to avoid emotional exhaustion due to surface acting:<br><br>**1. Instrumental support**: can signal willingness of organization to deliver material (PPE, kit) and resources (social distancing measures, disinfected wards) to foster nurses' capabilities to tackle increased job demands; intervention: provide protective equipment (i.e., mask, gloves and glass shield), sanitation, test kits and social distancing inside the ward<br><br>**2. Coaching Leadership** as human development process to promote desirable and sustainable change to the benefit of coachee and potentially all stakeholders (including structured, focused interaction; appropriate strategies, tools, and techniques); intervention: coaching leadership led by direct supervisor; creating positive and supportive workplace environment by helping nurses to reduce and cope with stress or resolve conflict among staff.	no	no	Implement-tation of interventions supervised by volunteers to maintain standards throughout the intervention phase (July-November 2021)

Inter-vention	Setting/ context	Work stressor/ rationale	Prevention approach	Modified work system components	Duration, materials, procedures, mode of delivery	Tailoring	Modifica-tions	Fidelity
		b) coaching leadership lessens the impact of job stress in emergency on emotional exhaustion (create positive and supportive workplace environment, help nurses to reduce and cope with stress, or resolve conflict among staff)						
**Triggered Palliative Medicine Consults in the Medical Intensive Care Unit** (Piscitello et al., 2022)	during COVID-19 surge<br><br>ICU nurses at one medical center	Moral distress likely increased for inpatient nurses caring for patients during the COVID-19 pandemic, e.g. through "futile" care or care that is not in line with the patient's wishes	primary	time<br>physical environment<br>resources<br>**X activities**<br>social	**Job-oriented approach**<br>each patient admitted to two geographically distant ICUs (one for Intervention group, one for concurrent Control group) between November 9 and December 18, 2020 between Monday and Friday;<br><br>**intervention trigger**<br>Attending physicians were contacted each morning to inform them of patients meeting at least one of the trigger criteria (e.g., admission from a skilled nursing or long-term care facility, or critical diagnosis.). Physicians could agree or disagree to placing a palliative medicine	unclear, possible due to visitor regulations (used as explanation for statistically nonsignificant reduction in nurses' moral distress)	unclear (possible arrangement of web meetings or telephone calls with patients' family members)	no

Inter-vention	Setting/ context	Work stressor/ rationale	Prevention approach	Modified work system components	Duration, materials, procedures, mode of delivery	Tailoring	Modifica-tions	Fidelity
		→ **aim/ hypothesis:** increased palliative medicine consults & scheduled family meetings for critically ill patients may be associated with less moral distress of ICU nurses during COVID-19 pandemic			consult. Control group: medical consults after standard care.<br><br>**Triggered consult** (Intervention): Patient must be seen by medicine clinician within 24hr of ICU admission.<br><br>**Arranged family meeting**: early family meeting by day 3 of admission with a repeat every 5-7 days while at the ICU; meeting needed to include discussion about patient preferences and values for medical care			
**proactive organizational approach** (Zaghini et al., 2021)	frontline COVID-19 nurses in specialized SARS-CoV-2 hospital	**workloads** and **changed requirements** negatively affect nurses' work-related stress, and consequently on their job satisfaction and quality of life<br><br>→ **aim/ hypothesis:** proactive organizational approach limits nurses' work-	Primary, secondary	time<br>**X physical environment**<br>**X resources**<br>**X activities**<br>**X social**	**Multi-level intervention**<br>**A) Job-oriented approaches:**<br>**1. nurse environment**<br>- reorganized structure of wards (e.g., increasing intensive care beds, establishing dedicated paths in the emergency rooms for immediate access to care)<br>- reorganized procedures (e.g., procedure for admitting suspected and positive patient in the Accident and Emergency, cleaning and disinfection wards and patients' unit or COVID-19 respiratory care management)<br>- reorganized internal paths within the separation of COVID-19 positive and negative patients	no	no	no

Inter-vention	Setting/ context	Work stressor/ rationale	Prevention approach	Modified work system components	Duration, materials, procedures, mode of delivery	Tailoring	Modifica-tions	Fidelity
		related stress level, and helps preserve job satisfaction and quality of life during SARS-CoV-2 pandemic			**2. nurse staffing and workload**<br>- nurse-patient ratio before Pandemic: medium care 1:9, high-intensity 1:4 → After transformation to COVID-19 ward redistributed staff to adequate staffing levels maintaining a ratio of nurses to patients;<br>- over 24 hours, ratio of 1:6 in SARS-CoV-2 units with medium care intensity and 1:2 in high-intensity units<br>**3. Participatory approach and autonomy**<br>- promoted through continuous clinical and organizational audits, lectures, and workshops; opportunities to discuss care adjustments<br>**4. Healthcare surveillance of nurses**<br>- staff exposed to Sars-CoV-2 kept under observation with nasopharyngeal swabs and successive serological samples<br><br>**B) person-oriented approaches**:<br>**1. Competence and learning motivation (in person/online)**<br>- training on the correct use of individual protection devices<br>- development of specific online tool (intranet) to distribute information material on SARS-CoV-2 (reporting guidelines, pathways, updated information issued by Italian government)<br>**2. process-focused unit level intervention**			

Inter-vention	Setting/ context	Work stressor/ rationale	Prevention approach	Modified work system components	Duration, materials, procedures, mode of delivery	Tailoring	Modifica-tions	Fidelity
					- organizational support measures including training, involvement, enhancement of skills and psychological support;<br>- psychological help desk for staff available every day on-site and remotely			
**Holistic sleep improvement strategies** (Y. Zhang et al., 2023)	frontline COVID-19 nurses in specialized SARS-CoV-2 hospital	**workloads** that affect nurses' sleep quality negatively<br><br>→ holistic sleep improvement strategies improve sleep quality of the frontline nurses	primary, secondary	X **time**<br>X **physical environment**<br>X **resources**<br>X **activities**<br>X **social**	**Multi-level intervention**<br>**A) Job-oriented approach:**<br>**1. scientific human resource management**:<br>- increased number of shifts, reducing working hours of each shift, keeping the workload under control to ensure sufficient rest or sleep after each shift<br>- rotation planning: avoid continuous rotation through reserved charges of nurses for rotation<br>- standardized pre-service training about COVID-19 pandemic<br>**2. comfortable sleep environment**<br>- quiet, comfortable hotel rooms near hospital for rest and sleep<br>- provided materials: thermal insulation products (electric blankets, hot water bags), goggles, earplugs to reduce light and noise interference,<br>- materials provided by: nursing managers (should pay attention to the temperature)<br>**2. humanistic care**<br>- positive feedback (timely affirmation, encouragement, acknowledgement)<br>- encourage nurses to keep in touch with family and friends	nurses could choose which courses to attend, or which consultation offers to use	no	no

Inter-vention	Setting/ context	Work stressor/ rationale	Prevention approach	Modified work system components	Duration, materials, procedures, mode of delivery	Tailoring	Modifica-tions	Fidelity
					- organizing initiatives to solve living difficulties of nurses' families (e.g., providing especially nurses' children with food, books and surgical masks), provided by: logistics supports department<br><br>**B) person-oriented approach**<br>**1. self-relaxation and self-adjustment training**:<br>- respiratory relaxation and progressive muscle relaxation courses could be attended twice a week, provided by rehabilitation therapists (could be delivered online)<br>- provided materials: white noise and music packages to listen before sleep<br>- set up sleep department (free access for nurses) for consultation by experts specialized on sleep<br>- materials for sleep department: different therapy and relaxation devices (e.g., for transcranial magnetic stimulation therapy)<br>**2. humanistic care**<br>- psychological assessment at the beginning and end of frontline work; availability of counselling and assistance every day, provided by: psychologists (could be delivered online)<br><br>other collaborators: sleep improvement collaboration team including hospital administrators			

Inter-vention	Setting/ context	Work stressor/ rationale	Prevention approach	Modified work system components	Duration, materials, procedures, mode of delivery	Tailoring	Modifica-tions	Fidelity
**Work break organization**								
**Motivational Messages Sent to Emergency Nurses** (Goktas et al., 2022)	emergency nurses working dayshifts in emergency department of two hospitals	increased workload, longer working hours, isolation from family, and limited resources for inpatient nurses promoted adverse physical, social and psychological factors of nurses<br><br>→ a**im/ hypothesis:** motivational messages enhance nurses' job satisfaction, decrease their compassion fatigue, and favorably affect their communication skills	secondary	**X time** physical environment resources **X activities** social	**Multi-level intervention**<br>**A) person-oriented part of intervention:**<br>nurses received three motivational messages per day (7am: "good morning", 12 pm: "health-promotion", 4 pm: "Me time") for 21 days (different messages each day) via their private smartphone<br><br>**B) job-oriented part of intervention**<br>nurses were allowed a 5-10 min break by the head nurse to check the messages; (messages were reviewed by 4 nurse academicians and<br>2 psychologists then pre-tested; content validity of 96%)	no	no	possible deviations from the intended intervention (participant forgetting phone, no allowance of head nurse to read messages, no time or other clinical priorities)

Inter-vention	Setting/ context	Work stressor/ rationale	Prevention approach	Modified work system components	Duration, materials, procedures, mode of delivery	Tailoring	Modifica-tions	Fidelity
**Resilience Bundle for Emergency Nurses** (Haugland et al., 2023)	during COVID-19 surge<br><br>emergency nurses of level I trauma center with >30% COVID-19 patients	Unit specific: understaffing (10% of the time), high turnover rates, increasing workplace stress, the emergency department had no strategies to improve resilience of employed nurses<br><br>→ aim/ **hypothesis:** enhancing the resilience of emergency nurses increases their joy in the workplace	secondary	time<br>**X physical environment**<br>**X resources**<br>**X activities**<br>social	**Multi-level intervention**<br>**A) Job-oriented approach**<br>**1.Serenity room:** quiet space for taking breaks to promote self-care, replenishment, reduce fatigue, and show employer's support/acknowledgement; *Materials*: massage chair, Vacant or occupied sign, aroma diffuser, LED candles, Sound machine, fluorescent light cover, tranquil tapestry or wall art<br><br>**2. Structured debriefing**: carried out by charge nurse (trained pre-intervention by study)<br>After a critical care event (death on the unit, highly stressful event, or at nurse's discretion) for involved interprofessional team;<br>goal: Build resilience by helping to understand emotions and experiences; *Materials*: STOP 5 debriefing form "Is everyone okay?" with four parts: Summarize the event, Things that went well, Opportunities for improvement, Points for action<br><br>**B) person-oriented approach**<br>**1. Relaxation and mindfulness program**:<br>carried out by charge nurse (trained pre-intervention by study)	nurses could chose when to use the serenity room; debriefing possible at nurse's discretion	unclear, possible due to charge nurse turnover, training of new charge nurses was needed	inability to measure scores for the same individuals across the surveys; <u>reasons</u>: staffing challenges (almost 26% of nurses' sick leaves; nurse-to-patient ration from 1:3 to 1:4, high turnover rates of >30%) high turnover (>46%) of charge nurses (who carried out debriefing/ mindfulness sessions)

Inter-vention	Setting/ context	Work stressor/ rationale	Prevention approach	Modified work system components	Duration, materials, procedures, mode of delivery	Tailoring	Modifica-tions	Fidelity
					Intervention activities: quiet deep breathing, journaling, or coloring; techniques were added to daily shift huddles and carried out by charge nurses; M*aterials*: Charge nurse received a copy two books for mindfulness techniques; Posters teaching and encouraging mindfulness were distributed throughout the unit.			
**Virtual Reality Relaxation** (Nijland et al., 2021)	during COVID-19 surge<br><br>ICU nurses working with COVID-19 patients at one hospital	high levels of stress during the COVID-19 pandemic<br><br>→ **aim/ hypothesis:** using VRelax during 10-min breaks would reduce experienced stress immediately	secondary	**X time**<br>**X physical environment**<br>**X resources**<br>**X activities** social	**Job-oriented approach**<br>Over a duration of 3 months VR sessions provided in a separate room during work shifts on COVID-19 wards (availability also evening and night); Recommended minimum time of use 10 min; nurses were encouraged to use VRelax (by trained (para)medical students/ by their team leaders) as short break during their shift; trained (para)medical students explained the use of VRelax<br><br>**VR-Relaxation Room**: separate room on ICU unit; head-mounted device (VR Oculus Go); comfortable swivel armchair; Hygiene precautions (hand hygiene, surgical caps during use, hydrogen peroxide wipes as disinfectant)	nurses could decide what environment or interactive elements to use and how long or often they used the Vrelax	no	barrier to use the intervention: high workload; Risk of nausea or dizziness when using VR

Inter-vention	Setting/ context	Work stressor/ rationale	Prevention approach	Modified work system components	Duration, materials, procedures, mode of delivery	Tailoring	Modifica-tions	Fidelity
					**Procedure**: Use of VR was explained before use; During use: Nurses could navigate through high quality immersive 360 degree natural environments (walking on the beach, under water swimming with dolphins, …); interactive elements were embedded (e.g., game of popping underwater air-bubbles, shooting start in a night sky, or audio tracks of relaxation exercise); after use devices were disinfected;			
**Use of "serenity lounges"** (Pagador et al., 2022)	during COVID-19 surge<br><br>registered nurses in 10 inpatients units of one medical center	during the COVID-19 pandemic, statistically significant increase in burnout and anxiety levels, emotional stress, turnover intentions and associated costs<br>→ **aim/ hypothesis:** evaluate use of serenity lounge and massage chair during the pandemic to reduce nursing staff's anxiety, stress, burnout	secondary	time<br>**X physical environment**<br>**X resources**<br>activities<br>social	**Job-oriented approach**<br>daily access to serenity lounges over 7 months (included a spike in admitted COVID-19 patients between December 2020 and February 2021)<br><br>**Serenity room with calm interior**: Old meditation rooms for patients' families were reused as serenity rooms for nurses;<br>materials: Calming wall color, Zen-themed wall decoration (paintings of nature, inspirational quotes), Himalayan salt-lamp, two recliners, electric percussion massagers, massage chair, tables, aroma diffuser, audio-system for nature sounds (e.g., waves, room could be darkened<br>**room use**: Nurses could book a slot via list (first-come-first serve); before use nurses notified head nurse or nursing assistant who then would assist with patient care and safety measures while the nurse is gone (aim: reduce	rooms were accessible every day; nurses could decide when and how long to use the room and/or the massage chair	no	used by 8-10 nurses per 12-hr-shift

Inter-vention	Setting/ context	Work stressor/ rationale	Prevention approach	Modified work system components	Duration, materials, procedures, mode of delivery	Tailoring	Modifica-tions	Fidelity
					interruptions); nurse would give hand-off to charge nurse, assistant			
**Healing Touch Intervention** (Rosamond et al., 2023)	inpatient nurses in acute care, critical care, perioperative care, inpatient dialysis care units	COVID-19 pandemic has **intensified nurse stress** in clinical environments<br><br>→ **aim/ hypothesis:** the positive intention and gentle hand movements of the Healing Touch practitioner balances and changes the recipient's biofield energy and decreases stress perception and physiological response	secondary	**X time** physical environment resources **X activities** social	**Job-oriented approach**<br>- pre-intervention: distribution of Flyers in the unit<br>- Intervention during 12-hr day work shift within a work break of 15 min (4-7 min Healing Touch session); setting: quiet room; delivered by Certified Healing Touch Practitioner<br>- Materials: technique and Healing Touch program student handbook	No	No	no

Supplement D

Table S5. *RoB-2*

Risk of bias for randomized controlled studies (conducted with RoB-2, Sterne et al., 2019) from least to most concerns

			**Risk of bias rated with RoB-2 (2019)**					
**First author**	**Publi-cation year**	**Intervention (brief name)**	**Randomiza-tion**	**Deviations from intended intervention**	**Missing outcome data**	**Measurement of the outcome**	**Selection of reported results**	**Overall**
Chang, Y. C.	2022	Integrated workplace violence management intervention	Some	Some	Low	Some	Low	Some
Goktas, S.	2022	Motivational messages	Low	Some	Low	Some	Low	Some
Rosamond, R.	2023	Healing Touch intervention *subjective* outcomes	Low	Some	low	High	Some	High
		objective outcomes	Low	Some	low	Some	Some	

Note. Risk of bias (RoB-2, 2019) = low / some / high concerns; Assessment was conducted for each study outcome. To offer a quick overview in this table, congruent ratings (e.g., for all subjective outcomes of a study) are depicted as one. The ranking from least to most concerns first follows the overall rating and the alphabetical order second.

Table S6. *ROBINS- I*

Risk of bias assessment for quasi-/nonrandomized studies (ROBINS-I, Sterne et al., 2016) from least to most concerns

		Risk of Bias rated with ROBINS-I (2016)								
**First author**	**Publication year**	**Intervention (brief name)**	**Confounding**	**Selection of participants**	**Classification of interventions**	**Deviations from intended interventions**	**Missing data**	**Measurement of outcomes**	**Reported result**	**Overall**
Kumar,	2022	Instrumental support and coaching leadership	moderate	moderate	low	low	low	moderate	moderate	moderate
Piscitello, G.M.	2022	Triggered palliative medicine consults								
		subjective (nurse) outcomes	moderate	moderate	low	moderate	moderate	moderate	moderate	moderate
		objective (patient) outcomes	low	low	low	low	low	low	moderate	
Golds-worthy, S.	2021	Professional development intervention simulation	moderate	low	low	low	serious	moderate	moderate	serious
Hung, C. L.	2023	Aroma therapy on wards								
		subjective outcomes	serious	low	low	low	serious	serious	moderate	serious
		objective outcomes	serious	low	low	low	serious	moderate	moderate	serious
Nijland, J.	2021	Virtual reality relaxation	serious	moderate	low	low	low	serious	moderate	serious
Zaghini, F.	2021	Proactive organizational approach	serious	low	low	low	low	serious	moderate	serious
Zhang, Y.	2023	Holistic sleep improvement strategies	serious	low	low	low	low	serious	low	serious
Haugland, W. A.	2023	Resilience bundle for emergency nurses	serious	low	low	serious	**critical**	serious	moderate	**critical**
Pagador, F.	2022	Use of "serenity lounges"	moderate	**critical**	low	low	serious	moderate	moderate	**critical**

Note. Risk of bias (ROBINS-I, 2016) = low / moderate / serious / critical; Assessment was conducted for each study outcome. To offer a quick overview in this table, congruent ratings (e.g., for all subjective outcomes of a study) are depicted as one. The ranking from least to most concerns follows the overall rating first and the alphabetical order second.

Supplement E

Table S7. *Rationale of included studies (pandemic associated work stressors and strains)*

Intervention (brief name)	Rationale: Pandemic associated…	
	Adverse work stressors	Adverse work strain/ consequences
Holistic sleep improvement strategies (Y. Zhang et al., 2023)	high *workload* and stress (adapt to new work tasks and ward, risk of infection and infecting others, direct patient contact, work hours, taking night shifts)	poor sleep quality
Proactive organizational approach (Zaghini et al., 2021)	*workload* and stress (*understaffing*, interpersonal conflicts, lack of autonomy)	less job satisfaction, Quality of life
Healing Touch intervention (Rosamond et al., 2023)	-	increased distress and burnout, poor quality of patient care, financial loss for institution
Triggered palliative medicine consults in the medical intensive care unit (Piscitello et al., 2022)	*workload* (not enough time for patients)	increased moral distress
Use of "serenity lounges" (Pagador et al., 2022)	-	increase in burnout levels, high levels of anxiety, emotional stress, turnover intentions and associated costs
Virtual reality relaxation (Nijland et al., 2021)	-	increased psychological stress, burnout
Instrumental support and coaching leadership (Kumar & Jin, 2022)	increased job demands require inpatient nurses to perform emotional labour	increased job stress, emotional exhaustion
Aromatherapy on ward (Hung et al., 2023)	*workload* due to *limited medical resources*, concerns over infection	severe anxiety, stress, and post-traumatic stress, disorder symptoms of nursing staff
Resilience bundle for emergency nurses (Haugland et al., 2023)	inadequate personal protective equipment, increased patient-to-nurse ratios, and higher acuity patients, Unit specific: *understaffing*	increased workplace stress, compassion fatigue, burnout; Unit specific: high turnover rates
Professional development simulation intervention (Goldsworthy, 2021)	*understaffing*	increased mental health issues, high turnover, turnover costs
Motivational messages sent to emergency nurses (Goktas et al., 2022)	*workload*, work hours, isolation, *limited medical resources*	poor job satisfaction and communication skills; increased compassion fatigue
Integrated workplace violence management (Chang et al., 2022)	workplace violence: higher rates of physical and verbal abuse	physical and mental health problems

Note. Written in *italics = work stressors that were given as rationale more than once*

Supplement F

Table S8. *Table of Outcomes: Rest break organization*

For formatting reasons, please refer to this section for an explanation of abbreviations in the columns:

- *First author*: Studies are alphabetically sorted after first author.
- *Study population*: IG = intervention group, CG = control group
- *Study design*: T refers to the time point of measurement (T0 = baseline before intervention, T1 = next measurement)
- *Significance and direction of outcomes*: ↑ = increased, ↓ = decreased; The *p* value indicates a statistically significant difference (e.g., between the IG and CG / before and after). In line with the APA 7th Edition guidelines, the exact p value is depicted (if given by the included study), unless p was < 0.001.
- *Overall quality assessment*: Includes the risk of bias domains (ROBINS-I and RoB-2) highlighted with **bold** letters in addition to other strengths (marked with +) and weaknesses (marked with -).

First author	Publication year	Country	Study population	Study design	Intervention (brief name)	Time frame, frequency/duration	Statistical significance and direction of outcomes	Statistically nonsignificant outcomes	Overall quality assessment
Goktas, S.	2022	Turkey	Emergency nurses of two designated pandemic hospitals (53.5% female) working only day shifts<br><br>IG n = 33<br>CG n = 32 (no intervention)<br><br>7.6% dropout rate	Randomized-controlled experimental study<br><br>(T0) pre intervention<br><br>(T1) after the 21 days of intervention	**Motivational Messages Sent to Emergency Nurses** (short break triggered by messages)	Over a duration of 21 days (July-August 2021), nurses received three motivational messages per day and would take 5-10 min breaks to check them	Over group and time:<br>↑job-satisfaction ($p < .05$)<br>↑communication skills ($p < .05$)<br><br>↓lower compassion fatigue ($p < .05$)		+ study design (RCT)<br>+ prespecified hypotheses tested<br>+ low concern for **selection of participants**<br>+ low concerns for **missing outcome data**<br>+ low concerns for **selection of reported results**: Study protocol registered and accessible<br>+ statistical power > .95 for effect size of .613 was reached<br><br>- some concerns for **deviation from intervention**: Unknown extent of participants forgetting their phone, unable to read messages, no permission, or having no time → no adherence measured<br>- some concerns for **measurement of outcomes**: Self-reported while knowing of the intervention
Haugland, W. A.	2023	USA	Emergency nurses (89.6% female) of level I trauma center with >30% of COVID-19 patients<br><br>IG n = 47<br><br>loss-to-follow up rate of 51.06%	Mixed methods pre-post-test design:<br><br>(T0) data collection at baseline<br><br>(T1) data collection 6 weeks after implementation of complete bundle	**Resilience bundle for emergency nurses** ("serenity room", structured debriefing, relaxation and mindfulness)	Conducted in 2021, 15 weeks with an implemented daily practiced resilience bundle	↑self-reported resilience (T1) compared to baseline ($p = .003$)	Perceived stress score<br><br>self-reported resilience (T2) compared to baseline	+ low concerns for **selection of participants**<br>+ low concerns for **classification of intervention**<br><br>- serious concerns for **confounding**: No confounds (e.g., adverse events over time, change in workload) measured, and no control group with similar characteristics<br>- serious concerns for **deviations from intended intervention**: Implementation success or adherence was not measured

First author	Publi-cation year	Country	Study population	Study design	Intervention (brief name)	Time frame, frequency/duration	Statistical significance and direction of outcomes	Statistically nonsignificant outcomes	Overall quality assessment
				(T2) data collection 15 weeks after implementation					(e.g., important for debriefing: how often conducted?) therefore unclear, how high charge nurse (who conducted the intervention) turnovers affected the adherence to debriefs and mindfulness exercises<br>- critical risk of bias for **missing data**: It was not possible to guarantee that the same participant was measured twice, there were certainly missing data per participant<br>- serious concern for **measurement of outcomes**: Self-report and knowledge of intervention<br>- moderate concerns for **selection of reported result**: No clear evidence that all reported results correspond to intended outcomes (no study protocol/preregistration)<br>- hypotheses of effects only implicitly stated<br>- no statistical power calculated
Rosa-mond, R.	2023	USA	Inpatient nurses (93% female) of various units (acute care, critical care, perioperative care, inpatient dialysis care) from different hospitals	Mixed-method cluster randomized controlled trial with matched pairs randomization<br><br>(T0) baseline self-report data and objective measurements	**Healing Touch intervention** during additional breaks	Conducted in February 2020; during 12-hr day work shift within a work break of 15 min (4-7 min Healing Touch session)	Subjective measurements<br>↑stress symptoms post treatment (T1; $p < .001$) and follow-up (T2; $p = .014$) compared to control group	(T1 and T2) heart rate, systolic blood pressure<br>(T1) respiratory rate	+low concern for **randomization**: Cluster randomized units in matched pairs<br>+low concern for **missing outcome data**<br>+reached power of 0.07 for medium effect size<br>- some concerns for **deviations from intended intervention**: Deviation in trials not reported<br>- matched pair randomization: unclear what criteria were

First author	Publi-cation year	Country	Study population	Study design	Intervention (brief name)	Time frame, frequency/duration	Statistical significance and direction of outcomes	Statistically nonsignificant outcomes	Overall quality assessment
			IG n = 75<br><br>CG n = 75 (deep breathing group)	(T1) one minute post-intervention second set self-reported data and objective measurements, three open-ended questions<br><br>(T2) follow-up after 1-4 hr. Third set of objective measurements and self-report			objective measurements<br>↑respiratory rate at follow-up (T2; $p < .001$) compared to control group		matched; Was the workload (possible confound) of units comparable?<br>- some concerns for **selection of reported result**: Heart rate, systolic blood pressure and respiratory rate were measured as indicators for "biofield energy". In reported results only respiratory rate was significantly (statistically) lower and only then interpreted as indicator for "improved stress levels" (p. 6)<br>- high concerns for **measurement of subjective outcome**: High probability of social desirability (scale filled out in front of Healing Touch practitioner)<br>- Some concerns for **measurement of objective outcome**: unlikely influence of knowledge of intervention on physical measures<br>- untrue statement in discussion: "found a statistically significant decrease in RR immediately post-intervention" (p.8)<br>- no hypotheses about physical measures and psychological measures stated before the analysis
Pagador, F.	2022	USA	registered nurses (84.6% female) across 10 inpatient units of one	pre-post-test design:<br><br>(T0) before use of serenity room	**Use of "serenity lounges"**	since November 2020; daily access to serenity lounges over 7 months (included a spike in admitted COVID-19 patients between	Use of serenity lounge:<br>↓less feelings of emotional exhaustion, burnout, frustration, being worn	no sign. higher reduction in emotional exhaustion, burnout, frustration, being worn out, stress,	+low concern for bias in **classification of intervention**<br>+low concerns for **deviations from intended intervention**<br>+overall aim/hypothesis tested<br><br>- moderate concerns for **confounding**: Workload as

First author	Publi-cation year	Country	Study population	Study design	Intervention (brief name)	Time frame, frequency/duration	Statistical significance and direction of outcomes	Statistically nonsignificant outcomes	Overall quality assessment
			medical center<br><br>IG n = 67<br><br>22.39% dropout rate	(T1) immediately after use of serenity room		December 2020 and February 2021)<br><br>evaluation is ongoing	out, stress, anxiety ($p < .001$) compared to before use<br><br>duration of massage chair use:<br>↓for 10-20 min than <10 min: higher reduction in feeling worn out ($p = .03$), emotional exhaustion ($p = .04$), and anxiety ($p = 0.01$) compared to before<br>↓for <20 min than <10 min: higher reduction in anxiety ($p = .03$) compared to before	after >20 min of use	possible confound (probably no change before to after use of the room but self-selection of participants); no control-group (change in outcomes due to pre-existing low workloads possible)<br>- **critical** risk of bias for **selection of participants**: Sample is very selected (self-selection; not all nurses used the room, and not all nurses filled out the questionnaire)<br>- serious concerns for **missing data**<br>- moderate concerns **for measurement of outcomes** (knowledge of intervention and self-assessment of outcomes)<br>- moderate risk for **selection of reported results**: No clear evidence that all reported results correspond to intended outcomes (no study protocol/preregistration)<br>- no systematic measurement of intervention use/adherence<br>- not marked explorative: no hypotheses about duration of use stated<br>- no statistical power calculated
Nijland, J.	2021	Nether-lands	ICU nurses (85% female) working with COVID-19 patients at one hospital	Pre-post-test design<br><br>(T0) immediately before use	**Virtual reality relaxation**	Over a duration of 3 months (May-June 2020, during the first wave of the COVID-19 pandemic) access to a separate room during work shifts	↓less perceived stress ($p < .005$) immediately after intervention compared to before		+low concern for **classification of intervention**<br>+low concern for **deviations from intervention**<br>+low concerns for **missing data**<br>+hypothesis/aim tested

First author	Publi-cation year	Country	Study population	Study design	Intervention (brief name)	Time frame, frequency/duration	Statistical significance and direction of outcomes	Statistically nonsignificant outcomes	Overall quality assessment
			IG n = 86<br><br>23.26% dropout rate	(T1) immediately after use<br><br>(T2) follow-up (questionnaires not comparable to T0 or T1)		on COVID-19 wards (also in the evening and night); with recommended time of use at least 10 min			- serious concerns for **confounding**: Possible self-selection, workload/ ratio of patients to nurse could influence opportunities to use the room (only 26% of all ICU nurses used VR), possible confounds tested with follow-up test (T2) retrospective perceived stress over intervention period for between user and non-user → Problem: only 138 of 326 nurses who had access filled out the follow-up questionnaire (response rate of 42.33%)<br>- moderate concerns for **selection of participants**: Possible self-selection due to workloads, differences in users- and non-users were assessed only retrospectively in follow-up (T2) but questionnaire had low-response rate<br>- serious concerns for **measurement of outcomes**: Self-report while knowledge of intervention; outcome only measured with one item (T1)<br>- moderate concerns for **selection of reported result**: No clear evidence that all reported results correspond to intended outcomes (no study protocol/preregistration)<br>- 86 nurses used VR; no information how many pre-post comparisons were calculated (nurses could attend multiple times)

First author	Publi-cation year	Country	Study population	Study design	Intervention (brief name)	Time frame, frequency/duration	Statistical significance and direction of outcomes	Statistically nonsignificant outcomes	Overall quality assessment
									- No statistical power calculated

Table S9. *Table of Outcomes: Further studies*

For formatting reasons, please refer to this section for an explanation of abbreviations in the columns:

- *First author*: Studies are alphabetically sorted after first author.
- *Study population*: IG = intervention group, CG = control group
- *Study design*: T refers to the time point of measurement (T0 = baseline before intervention, T1 = next measurement)
- *Significance and direction of outcomes*: ↑ = increased, ↓ = decreased; The *p* value indicates a statistically significant difference (e.g., between the IG and CG / before and after). In line with the APA 7th Edition guidelines, the exact p value is depicted (if given by the included study), unless p was < 0.001.
- *Overall quality assessment*: Includes the risk of bias domains (ROBINS-I and RoB-2) highlighted with **bold** letters in addition to other strengths (marked with +) and weaknesses (marked with -).

First author	Publi-cation year	Coun-try	Study population	Study design	Intervention (brief name)	Time frame, frequency/ duration	Statistical significance and direction of Outcomes	Statistically nonsignifica nt Outcomes	Overall quality assessment
Chang, Y. C.	2022	Taiwan	Emergency nurses of COVID-19 hospital (90.7% female)<br><br>IG n = 39<br><br>CG n = 36 (standard 1-hour in-service class only)	Cluster-randomized, pre- and post-test, controlled trial using parallel-groups<br><br>(T0) prior to intervention<br><br>(T1) after the intervention	**Integrated workplace violence management intervention** (organizational component were regular team-debriefings and feedback)	Conducted in 2020<br>12 sessions of at least 1h frequency/duration not reported	Over group and time:<br>↑ goal commitment (*p* < .001)<br>↑ occupational coping self-efficacy (*p* < .001)<br>↑ confidence in managing violence (*p* < .001)<br>↑ attitudes toward aggressive behavior and explanation of violence (*p* < .001)	Attitudes toward aggression in ED	+ study design (RCT)<br>+ low risk for **missing outcome data**<br>+ low risk for bias in **reporting results**: Study protocol conducted (but not accessible)<br>- some concerns for **randomization**<br>- some concerns due to **deviations from intended interventions**<br>- some concerns in **measurement of outcomes**<br>- unspecific hypothesis: "could exert synergistic effects on improvements in outcomes" (p. 3)<br>- statistical power of .80 for medium effect size of .25 not fully reached (n = 40 for each group)
Golds-worthy, S.	2021	Canada	Critical care unit nurses (89.5% female); both groups from different hospitals<br><br>IG n = 182<br><br>CG n = 181<br><br>overall dropout rate 61.99%	Quasiexperimental non-equivalent control group design with multiple points of measurement pre-post:<br><br>(T0) prior to the simulation portion of the intervention;<br><br>(T1) two weeks after	**Professional development intervention simulation** (new or changed work activities in practicum)	Over the course of one year period; 324-hr self-paced, critical care certificate program with Online Theory Component: 315h (6 courses) and Simulations intervention: 39h	Group differences at (T3) controlled by (T0) measurements:<br>↑ intent to stay in the unit in intervention group compared to control group (*p* = .02)<br>↑ intent to stay in the profession in intervention	Group difference at (T3) in intent to stay in the organization	+ prespecified hypotheses tested<br>+ low concerns for **selection of participants**<br>+ low concerns for **classification of intervention**<br>+ low concerns for **deviation from intervention**<br><br>- moderate concerns due to **confounding**: High attrition in the sample could be due to leaving the profession, remaining sample would then be those who are intent to stay<br>- serious concerns for **missing data** (high attrition of the sample): No indication how missing data was handled<br>- moderate concerns **for measurement of outcomes**: Self-report, knowledge of

First author	Publi-cation year	Coun-try	Study population	Study design	Intervention (brief name)	Time frame, frequency/ duration	Statistical significance and direction of Outcomes	Statistically nonsignificant Outcomes	Overall quality assessment
				(T0) and post-simulation<br><br>(T2) three months after (T1) and at the end of the practicum portion of the intervention;<br><br>(T3) 6 – 8 months after (T2) dependent on practicum completion time;<br><br>comparison group measured only at (T0) and (T3)		ICU preceptored clinical practicum over ten 12-hr-shifts (120h)	group compared to control group ($p < .001$)<br><br>mediator analysis for perceived organizational support<br>↑ direct effect: professional development intervention predicted higher intent to stay in the profession ($p < .05$)<br>↑ indirect effect: perceived organizational support mediated relationship between professional development and intent to stay in profession ($p < .05$)		intervention (unlikely to affect intent of stay)<br>- moderate concerns for **selection of reported result**: No clear evidence that all reported results correspond to intended outcomes (no study protocol/preregistration)<br>- no power calculated.

First author	Publi-cation year	Coun-try	Study population	Study design	Intervention (brief name)	Time frame, frequency/ duration	Statistical significance and direction of Outcomes	Statistically nonsignificant Outcomes	Overall quality assessment
Hung, C. L.	2023	Taiwan	Nursing staff (100% female) from different inpatient units (conven-ience sample)<br><br>IG n = 30<br><br>13.33% dropout rate	Pre-post-test design<br><br>(T0) prior to intervention<br><br>(T1) one day after four weeks of intervention	**Aromatherapy on ward**	For 4 weeks (during the second COVID-19 outbreak from April – June 2021) exposure to aroma diffused scent on ward twice every weekday (Friday-Monday) at 8:00-12:00 a.m. and 16:00-20:00 p.m.	Objective measurements: statistically significant change in physical stress indicators only for subgroups, e.g.:<br>↑ICU nurses' physical indicators for level of stress (activities of parasympathetic and sympathetic nervous system) higher after intervention compared to before ($p < .05$)<br><br>Subjective measurements<br>↓nurse stress: Work concerns ($p = .029$)<br><br>↓overall burnout score (= degree of fatigue; $p = .017$)	Physical stress indicators over all participants (heart rate variability)<br><br>nurse stress questionnaire overall<br><br>burnout score: client-related burnout	+low concern for **selection of participants**<br>+low concern for **classification of interventions**<br>+low concerns for **deviations from intended intervention**<br>+combination of objective and self-report measures<br>+"acceptable power to support the hypothesis" (p. 10)<br><br>- serious concerns for **confounding**: Different exposure to workloads in units could be a confound; no control group with similar characteristics (e.g., workload) that went without intervention<br>- serious concerns for **missing data**: 13.33% of participants did resign or were transferred to different unit (reason could be workload)<br>- serious concern for **measurement of** subjective **outcome** (knowledge of intervention and self-report)<br>- moderate concern for **measurement of** objective **outcomes**<br>- moderate concerns **for selection of reported result**: No clear evidence that all reported results correspond to intended outcomes (no study protocol/preregistration)<br>- analysis was mostly explorative (no hypotheses stated before analysis): thereby facets or subgroups were found to be statistically significant (not stated before data analysis in hypotheses)<br>- no statistical power calculated

First author	Publi-cation year	Coun-try	Study population	Study design	Intervention (brief name)	Time frame, frequency/ duration	Statistical significance and direction of Outcomes	Statistically nonsignifica nt Outcomes	Overall quality assessment
Kumar, N.	2022	Pakis-tan	COVID-19 frontline nurses (ICU) of 107 government hospitals (41.7% female) working 12h shifts (conven-ience sample)<br><br>IG n = 319	Pre-post-test design<br><br>(T0) before the intervention at three times of the day (morning: emotional labor, afternoon: job stress in emergencies, evening: emotional exhaustion)<br><br>(T1) after 3 months interval: self-reported outcomes on three consecutive days (Day 1: instrumental support, Day 2: supervisor's coaching leadership and job stress, Day 3: emotional exhaustion)	**Instrumental support and coaching leadership**	Started July 2020; provision of resources was followed by 3 months interval	↓instrumental support alleviates the undesirable effect of emotional labor on job stress ($p < .001$)<br>↓coaching leadership lessens the undesirable effect of job stress on emotional exhaustion ($p < .001$)		+low concern for **classification of interventions**<br>+low concerns for **deviations from intended intervention**<br>+low concerns for **missing data**<br>+specific hypotheses tested<br><br>- moderate concern for **selection of participants**: Possible self-selection of eligible participants before intervention due to job stress (?)<br>- moderate concerns for **confounding**: No controls in analysis; workload of nurses may have varied over time affecting perceived job stress. Not controlled for.<br>- moderate concerns for **measurement of outcomes**: self-reported outcomes while knowledge of intervention<br>- moderate concerns for **selection of reported result**: No clear evidence that all reported results correspond to intended outcomes (no study protocol/preregistration)<br>- no statistical power calculated
Pisci-tello, G. M.	2022	USA	ICU unit nurses at one hospital	Pre-post-test design<br><br>(T0) within 3 weeks pre	**Triggered palliative medicine consults** in medical	For 6 weeks (during the height of the second wave of the	Primary nurse outcomes:<br>↓nurse turnover intention due	Primary nurse outcome: pre-post difference of	+low concern for **classification of interventions**<br>+inclusion of **patient outcomes** (which could affect nurses' moral distress) compared to control group

First author	Publi-cation year	Coun-try	Study population	Study design	Intervention (brief name)	Time frame, frequency/ duration	Statistical significance and direction of Outcomes	Statistically nonsignificant Outcomes	Overall quality assessment
			nurses:<br>IG n = 48<br>20% dropout rate<br><br>patients:<br>IG n = 50<br>CG n = 57	intervention<br>self-report moral distress survey;<br><br>(T1) within 3 weeks after intervention	intensive care unit	COVID-19 pandemic), continuous checks if patients met criteria for triggered palliative medicine consults; Consults must be seen within 24h of ICU admission; criteria of family visits must be met by day 3 of admission and evaluated every 5-7 days	to moral distress (p = .006)<br><br>secondary patient outcomes:<br>↓rate of documented alternate decision makers (p < .001)<br>↓discharge rate to facility or hospice (p < .001)<br><br>↓time to transition to do not resuscitate status (p = .029)<br>↓days from ICU admission to palliative consult (p < .001)<br>↓patient costs for specific subgroups lower than in control group (e.g., p = .003 for patients with do not	moral distress<br><br>secondary patient outcomes: overall costs per patient in intervention compared to control group; rate of do not resuscitate code status in intervention vs. control group; no decrease in median ICU stay	+reporting of hindrances to intervention<br>+(for patients) statistical power of .80 reached to detect absolute difference of 27% between groups<br><br>- moderate concerns for **confounding**: Intervention was for high-risk patients. Nurses had other patients on the side (which with high certainty increased during COVID-19 surge probably contributing moral distress)<br>- moderate concern for **selection of participants**: Possible self-selection? All n = 78 nurses took part in the intervention. Return of questionnaires was only 62% pre-intervention and 42% in the post-intervention survey<br>- moderate concerns for **deviations from intervention**: COVID-19 regulations affected family visits → digital visits had to be arranged<br>- moderate concerns for **missing data**: 20% drop-out rate<br>- moderate concerns for **measurement of outcomes** (self-reports)<br>- moderate concerns for **selection of reported result**: No clear evidence that all reported results correspond to intended outcomes (no study protocol/preregistration)<br>- no hypotheses for specific outcomes stated before analysis<br>- statistical power only calculated for patient outcomes

First author	Publi-cation year	Coun-try	Study population	Study design	Intervention (brief name)	Time frame, frequency/ duration	Statistical significance and direction of Outcomes	Statistically nonsignifica nt Outcomes	Overall quality assessment
							resuscitate order)		
Zaghini , F.	2021	Italy	Frontline COVID-19 nurses (75.5% female) of COVID-19 hospital (convenienc e sample)<br><br>IG n = 350<br><br>8% drop-out	Mixed methods one group pre-post-test design:<br><br>(T0) self-report data collection (February 2020), 6 focus groups for qualitative data collection.<br><br>(T1) self-report data collection (July 2020)	**Proactive organizational approach** (nurse environment, nurse staffing, workload, competence and learning motivation, participation, autonomy, process-focused unit-level intervention, healthcare surveillance)	Proactive planning started after "patient zero" was identified with COVID-19 in Italy; 3 months of intervention from March 2020 to Mai 2020 (exponential increase of COVID-19 cases and lockdown in March)	Compared to baseline<br>↓job-related stress ($p < .001$)<br>↑job satisfaction ($p < .001$)<br>↑quality of life ($p = .003$)	Single facets of job-related stress, satisfaction, and quality of life	+low concerns for **selection of participants**<br>+low concerns for **classification of intervention**<br>+low concerns for **deviations from intervention**<br>+low concerns for **missing data**<br>+explicitly stated hypothesis<br>+statistical power: minimum sample size of 140 participants reached<br><br>- serious concerns for **confounding**: Workload Could influence, e.g., job-related stress; Although nurse-to-patient-ratio was stated to be held on a maximum of 1:6 in medium care and 1:2 in high intensity units it was not measured pre- and post how high the actual ratio was during time of measurement; Also, use of support offers (e.g., psychological help desk) not documented<br>- serious concerns for **measurement of outcome**: Nurses were aware of proposed outcome (job stress, job satisfaction, quality of life), and they self-assessed the outcome<br>- moderate concerns for **selection of reported result**: No clear evidence that all reported results correspond to intended outcomes (no study protocol/preregistration)<br>- generalizability of results suffers due to convenience sampling

First author	Publi-cation year	Coun-try	Study population	Study design	Intervention (brief name)	Time frame, frequency/ duration	Statistical significance and direction of Outcomes	Statistically nonsignificant Outcomes	Overall quality assessment
Zhang, Y.	2023	China (Wu-han)	Frontline COVID-19 nurses (96.2% female) of COVID-19 hospital (convenience sample)<br><br>IG n = 52	One group pre-post-test design<br><br>(T0) at baseline<br>(T1) after 4-week follow-up	**Holistic sleep improvement strategies** (scientific human resource management, comfortable sleep environment, self-relaxation/-adjustment, humanistic care)	Conducted in February 2020 the implemented strategies were practiced for 4 weeks	↑ Overall Sleep Quality Index compared to baseline (p = .004)<br><br>sleep quality facets compared to baseline:<br>↑subjective sleep quality (p = .016)<br>↑sleep efficiency (p = .015)<br>↓sleep disturbances (p = .007)	Sleep quality facets compared to baseline: sleep latency, sleep duration, sleep medication, daytime dysfunction	+low concerns for **selection of participants**<br>+low concerns for **classification of intervention**<br>+low concerns for **deviations from intervention**<br>+low concerns for **missing data** (no missing data/ loss to follow-up)<br>+low concerns for **selection of reported result**: Study protocol before intervention (not accessible)<br>+overall hypothesis tested<br>+statistical power of .90 reached for moderate effect size (required sample of 43 participants)<br><br>- serious risk for **confounding**: Effect cannot clearly be traced back to intervention (no control)<br>- serious risk for **measurement of outcomes**<br>- for facets no hypotheses were tested; might be explorative<br>- generalizability of results suffers due to convenience sampling (only 52 nurses in one single unit, convenience sample),<br>- no measurement of intervention use/adherence
